# Dual-Protein Intervention in CT26 Tumor-Bearing Mice: A Preliminary Evaluation of Its Effects on Anti-Tumor Efficacy of 5-Fluorouracil and Immune Responses

**DOI:** 10.3390/nu18111663

**Published:** 2026-05-22

**Authors:** Duo Feng, Mengjie Li, Di Han, Menghan Ma, Wenjuan Man, Na Li, Hu Li, Ruiqi Xu, Jiayu Fan, Jing Wang

**Affiliations:** 1 Institute of Food and Nutrition Development, Ministry of Agriculture and Rural Affairs, Beijing 100081, China; 15525926785@163.com (D.F.); limj0804@126.com (M.L.); handi87@126.com (D.H.); mmh0725@163.com (M.M.); m18252173489@163.com (W.M.); 15910807606@139.com (N.L.); lihu01@caas.cn (H.L.); 821012450672@caas.cn (R.X.); 19852835599@163.com (J.F.); 2 Special Food Research Institute, Qingdao Agricultural University, Qingdao 266109, China

**Keywords:** colorectal cancer, dual-protein, pre-intervention, nutrition intervention, combined treatment

## Abstract

Background: Colorectal cancer is a common malignancy and 5-fluorouracil (FU) remains a mainstay of chemotherapy despite its toxicity. As an important part of comprehensive tumor treatment, dual-protein (DP) nutritional intervention is attracting more and more attention. Methods: This study preliminarily evaluated the regulatory effects of DP intervention on colorectal cells of CT26 tumor-bearing mice, examining the dosage and administration methods of DP, as well as the anti-tumor effects of FU alone or in combination with DP. Results: The results showed that low- and medium-dose DP numerically increased spleen index and showed trends toward alleviating FU-induced thymic atrophy, splenic damage, nephrotoxicity, and myocardial injury. It also partly mitigated muscle wasting, prevented FU-induced shortening of the colorectal tract, and reduced intestinal injury. In addition, DP was associated with increased lymphocyte, monocyte, and platelet counts and decreased granulocytes, suggesting possible alleviation of chemotherapy-induced bone marrow suppression and a potential effect on hematopoietic function. Flow cytometry results indicated possible effects of DP on CD4^+^ T and CD8^+^ T cell proliferation or apoptosis, modulation of effector and memory phenotypes, reduced splenic neutrophil levels, balanced B cell function, and maintained natural killer cell activity. In addition, DP intervention also showed trends toward regulating hepatic lipid metabolism and partially alleviating FU-induced dyslipidemia and muscle damage. In addition, DP and FU could increase IL-2, IL-10, GM-CSF and IFN-γ and decrease IL-6 and TNF-α. Conclusions: In conclusion, a moderate dose (0.67 g/kg) of DP had the most favorable trends, and the pre-intervention mode was more effective. This study also provided exploratory data on the potential of DP in reducing chemotherapy-related toxicity. These findings will provide preliminary scientific support for nutritional therapy in colorectal cancer patients, as well as for the research, development, and application of dual-protein foods for special medical purposes.

## 1. Introduction

Colorectal cancer is a common malignant tumor of the digestive tract. A survey of global cancer statistics for 2022 found that cancer is a leading cause of death and a major obstacle to increasing life expectancy worldwide [1]. The development of colorectal cancer is closely linked to dietary nutrition, and optimizing protein intake is a key strategy in its comprehensive management [2]. Adequate diet and protein intake are very important for human health [3]. Compared with a single protein, a mixture enriched with 35% whey, 25% casein, 20% soybean protein and 20% pea had a more balanced plasma amino acid profile [4]. The identification of transcripts regulated by soy protein isolate (SPI) and whey protein hydrolysate (WPH) showed that the common and unique mechanism of anti-tumor action may involve growth factors, neuroendocrine and immune system genes. SPI and WPH could induce somatostatin, which can inhibit the occurrence of tumors [5]. In addition, a few rodent studies have shown that a high-protein diet could reduce 5-fluorouracil (FU) toxicity (casein) [6] or improve muscle function during chemotherapy (whey protein) [7], while another study examined protein intake effects on nutritional status [8]. However, all these studies used single-source proteins.

As a new compound nutritional formula, dual protein (DP) combines the complementary advantages of soybean protein from plants and whey protein from animals. Compared with a single-protein supplement, DP has a more balanced amino acid composition, higher nutrition utilization efficiency, and can avoid the nutritional imbalance caused by long-term single-protein intake. It also provides a range of benefits, including enhancing the body’s antioxidant capacity, improving intestinal barrier function, increasing skeletal muscle mass, and promoting hematopoiesis and immune system recovery. As such, it plays a crucial role in maintaining overall health and promoting growth and development [3,9].

In recent years, the concept of prehabilitation has gradually gained attention [10]. Although not yet standardized, it can be understood as a range of interventions initiated prior to surgery, with the core objective of enhancing patients’ physical, nutritional, medical, and psychological conditions. These interventions include nutritional interventions such as whey protein supplementation and the consumption of foods rich in protein and vitamin D, as well as personalized nutritional counseling. Some studies have even demonstrated that these interventions can reduce post-operative morbidity. However, the field remains in an experimental phase, and the optimal protocol has yet to be established, which limits the widespread promotion and clinical application of this concept [11]. Generally, an intervention period of 4 weeks or longer is considered sufficient to achieve physical and functional improvements, and older or frail patients may require a longer duration, but excessively prolonged interventions are not appropriate in the context of cancer [12]. It is important to clarify that the term “pre-habilitation” typically refers to interventions initiated after a disease has been diagnosed but before specific phases of treatment such as surgery or chemotherapy. In contrast, the nutritional intervention in the present study was administered before tumor inoculation, i.e., prior to disease onset. Therefore, it is more accurately described as a preventive nutritional intervention rather than pre-habilitation. Accordingly, we have adopted the term “pre-intervention” to describe the DP administration started 14 days before CT26 tumor cell injection. This preventive approach aims to improve the host’s nutritional and immune status before tumor establishment, thereby providing a clearer framework for evaluating the effects of early nutritional support.

In addition, chemotherapy drugs for colorectal cancer, such as FU, a classic nucleoside metabolism inhibitor, often cause side effects by interfering with DNA synthesis in rapidly proliferating cells. Combining nutritional intervention with chemotherapy may therefore alleviate these adverse reactions while improving metabolic and immune status, thereby enhancing overall therapeutic efficacy [13]. The DP used in this study, composed of soybean and whey protein, has been proved to be beneficial to human health, including improving severe blood disease [14,15], osteoporosis [9], atherosclerosis [16], diabetes [17] and so on. Therefore, we administered different doses of DP to Balb/c mice for the first time by gavage, followed by subcutaneous injection of CT26 colorectal cancer cells, and continued gavage throughout the period of tumor growth. The aim was to evaluate the tumor regulation effect of DP intervention, further explore its anti-tumor effect combined with FU, and analyze its potential immunological mechanisms.

## 2. Materials and Methods

### 2.1. Chemicals and Reagents

RPMI 1640 medium and penicillin–streptomycin solution were purchased from Procell Life Science & Technology Co., Ltd. (Wuhan, China). Fetal bovine serum (FBS) was obtained from Gibco (Grand Island, NY, USA) and 4% paraformaldehyde, xylene, and absolute ethanol were purchased from Sinopharm Chemical Reagent Co., Ltd. (Shanghai, China). Phosphate-buffered saline (PBS, pH 7.2–7.4) was obtained from ZSGB-BIO Co., Ltd. (Beijing, China). Hematoxylin staining solution and neutral balsam were purchased from Beijing Bioss Biomedical Technology Co., Ltd. (Beijing, China). Fluorouracil (FU) for injection was obtained from Tianjin King York Group Co., Ltd. (Tianjin, China). The blood biochemical assay kit was purchased from Jiangsu Zecheng Biotechnology Co., Ltd. (Taizhou, China). Human peripheral blood mononuclear cell separation medium (FICOLL-based) was obtained from Tianjin Hao Yang Biological Products Technology Co., Ltd. (Tianjin, China). Cyto-Fast™ Fix/Perm Buffer Set, Cell Activation Cocktail (with Brefeldin A), RBC Lysis Buffer (10×), and red blood cell lysis buffer were purchased from Biolegend (San Diego, CA, USA). RPMI 1640 medium was obtained from Beijing Aobing Biotechnology Co., Ltd. (Beijing, China). The triple antibiotic solution was purchased from Beyotime Institute of Biotechnology (Shanghai, China). FITC Hamster Anti-Mouse CD3e, APC Rat Anti-Mouse CD4, RB780 Rat Anti-Mouse CD8a, RB705 Rat Anti-Mouse CD45, PE-Cy7 Rat Anti-Mouse CD45R/B220, PE Hamster Anti-Mouse CD49b, FITC Rat Anti-CD11b, and APC Rat Anti-Mouse Ly-6G were obtained from BD Biosciences (San Jose, CA, USA). Fixable Viability Dye 740 was purchased from Hangzhou Star Biotechnology Co., Ltd. (Hangzhou, China). Bio-Plex Pro kit (12009159) was purchased from BIO-RAD (Hercules, CA, USA).

Dual-protein (DP) complex nutrition powder is light yellow, powdery, with no peculiar smell and no foreign bodies can be seen in normal vision. This product was made by our team in the early stage, please refer to the supplementary document for its nutritional profile and amino acid composition (Tables S1 and S2).

### 2.2. Animal Experiment

Eighty 4-week-old male SPF BALB/C mice, weighing 16–19 g, were purchased from Spefford (license number: SCXK (Beijing) 2024-0001, Beijing, China). The feeding environment was maintained at a temperature of 26 ± 1 °C and a humidity of 60 ± 5% with sterilized feed and water ad libitum. The experiment was carried out after one week of acclimatization. The entirety of animal experiments conducted received approval from the Animal Management and Ethics Committee of the Institutional Animal Care and Use Committee, Institute of Food and Nutrition Development, Ministry of Agriculture and Rural Affairs, as per the stipulated license number IFNDLLSC20250313-3.

### 2.3. Cell Culture

CT26.WT cells were cultured in RPMI 1640 complete medium supplemented with 10% fetal bovine serum (FBS) and 1% triple antibiotic solution (100 U/mL penicillin, 100 µg/mL streptomycin, and gentamicin) at 37 °C in a 5% CO_2_ cell incubator (Thermo Scientific, Waltham, MA, USA). Upon reaching 80–90% confluence, the cells were detached via enzymatic digestion. The digestion was terminated by adding complete RPMI 1640 medium when microscopic observation revealed cytoplasmic retraction, loss of intercellular connections, and a characteristic rounding up of the cells. Subsequently, the cells were subcultured at a split ratio of 1:2 to 1:4. Cells in the logarithmic growth phase, typically harvested 2–3 days post-seeding, were used for subsequent subculturing or experimental procedures.

### 2.4. Establishment of a Tumor-Bearing Mouse Model

CT26.WT cells in the logarithmic growth phase were harvested when they reached 90% confluence in the culture flask, resuspended in PBS at a concentration of 1 × 10^7^ cells/mL, and 100 μL of the cell suspension was inoculated subcutaneously into mice (hair was shaved before inoculation). This time point was designated as day 0 of model establishment (i.e., day 21 of the overall experimental timeline). Tumor nodules became palpable 3–5 days after inoculation. Tumor formation was confirmed when the tumor volume reached 100–120 mm^3^. On day 28 (i.e., 7 days after inoculation of CT26.WT cells), mice were randomly assigned into control and treatment groups using simple random sampling, and interventions were initiated. All mice were euthanized on day 45. During this period, the tumor induction rate was assessed and the mice were monitored for mental and activity status, tumor growth, coat color and appetite.

### 2.5. Animal Experimental Design and Mouse Grouping

**Figure 1:**
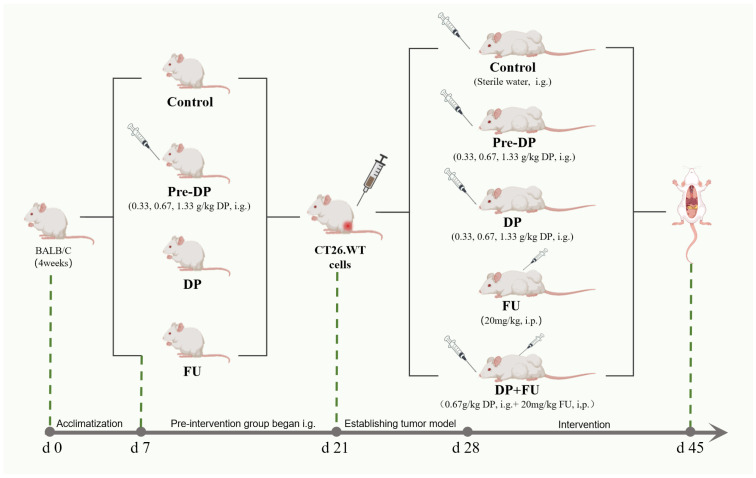
Flowchart of animal experiment design.

After 1 week of acclimatization, the mice were randomly divided into 10 groups using block randomization based on body weight and gavage was performed at a dose of 10 mL/kg. The control (CON) group received sterile water by i.g. starting from day 7. The DP doses were selected based on previous studies and studies of our team [16,17]. The pre-intervention group began receiving oral doses of DP (0.33, 0.67, 1.33 g/kg, i.g.) and, after 14 days of administration, a mouse tumor model was established. Seven days after inoculation with CT26.WT cells, the intervention group began oral administration of different doses of DP (0.33, 0.67, 1.33 g/kg, i.g.) for 17 consecutive days. The control group received normal saline by i.g. starting from day 7. The Pre.DP-L, Pre.DP-M, and Pre.DP-H groups received DP (i.g.) from day 7 until sacrifice at doses of 0.33, 0.67, and 1.33 g/kg, respectively. The DP-L, DP-M, and DP-H groups received the same respective doses of DP (i.g.) from day 28 (7 days post-modeling). The FU group (20 mg/kg/mouse, i.p., every other day) and the combination therapy group (DP by i.g. plus FU treatment) were also established. Each treatment group was housed in two separate cages (4 mice per cage) in this study. Only mice from the same treatment group were placed in the same cage. Mice in different treatment groups were not mixed to prevent cross-contamination. To reduce observation bias, group allocation and numbering were performed by a researcher who was not involved in the follow-up experiments. This person assigned a number and did not disclose the specific treatment details to the staff responsible for animal care or result measurement. The personnel in charge of daily observation, body weight measurement, tumor volume measurement, blood collection and euthanasia were blinded to the intervention of each group. For specific procedures, refer to Figure 1 (Flowchart of animal experiment design) and Table 1 (Dosage and experimental grouping). During this period, mouse body weight and tumor volume were measured every 2 days (Equation (1)).

To illustrate the nutritional impact of DP supplementation, we estimated the daily intake of selected nutrients from the maintenance feed and from the additional DP gavage (0.67 g/kg dose) using the value of 5 g/day feed intake for a 20 g male BALB/c mouse [18], under the isocaloric substitution assumption. The results are shown in Table 2.

(1) $$\mathrm{Tumor}\;\mathrm{volume}\left({\mathrm{mm}}^{3}\right)=\frac{\mathrm{length}\times {\mathrm{width}}^{2}}{2}$$

### 2.6. Organ Index, Colorectal Lengths and Tumor Inhibition Rate in Mice with Colorectal Tumor

After the last administration, the mice were weighed, and blood was collected from the retro-orbital sinus and the mice were euthanized by dislocation of the neck. Heart, thymus, liver, spleen, kidney, gastrocnemius, colon and rectum were dissected, and organ index was calculated according to Equation (2). After dissection, the length below the colon of mice was measured and the average value was calculated. In addition, the tumor inhibition rate was calculated according to Equation (3).

(2) $$\mathrm{Organ}\;\mathrm{index}\;\left(\%\right)=\frac{\mathrm{Organ}\;\mathrm{weight}}{\mathrm{Body}\;\mathrm{weight}}\times 100\%$$

(3) $$\mathrm{Tumor}\;\mathrm{inhibition}\;\mathrm{rate}\;\left(\%\right)=\frac{\mathrm{Average}\;\mathrm{tumor}\;\mathrm{weight}\;\mathrm{in}\;\mathrm{Control}\;\mathrm{group}-\mathrm{Average}\;\mathrm{tumor}\;\mathrm{weight}\;\mathrm{in}\;\mathrm{Treatment}\;\mathrm{groups}}{\mathrm{Average}\;\mathrm{tumor}\;\mathrm{weight}\;\mathrm{in}\;\mathrm{Control}\;\mathrm{group}}\times 100\%$$

### 2.7. Routine Blood Analysis of Mice

After orbital blood collection, 30–50 μL of whole blood was placed into the sample tube of a hematology analyzer (HF-3800, Beijing Halife Biotechnology Co., Ltd., Beijing, China). Following appropriate dilution, the analyzer was used to measure white blood cells (WBC), red blood cells (RBC), lymphocytes (LYM), hemoglobin (HGB), platelets (PLT), monocytes (MON) and granulocytes (GRA).

### 2.8. Detection of Spleen Tissue in Mice by Flow Cytometry

The spleen was aseptically removed and placed onto a 70 μm Falcon cell strainer (Corning Life Sciences, Durham, NC, USA) seated on a 50 mL centrifuge tube. The spleen was minced with sterile scissors, and the tissue was gently pressed through the strainer using the plunger of a syringe. The strainer was rinsed three times with 3 mL of PBS each to collect the cells thoroughly. The resulting cell suspension was centrifuged at 500× *g* for 5 min, and the supernatant was discarded. The residual liquid was gently agitated to loosen the cell pellet. Red blood cell lysis buffer (5 mL) was added, and the sample was vortexed for 30 s, then incubated on ice for 5 min with occasional agitation. The reaction was stopped by diluting the lysis buffer with 20–30 mL of 1× PBS, followed by centrifugation at 500× *g* for 5 min, after which the supernatant was removed. The cells were resuspended in 5 mL of complete 1640 medium to obtain a single-cell suspension. For surface antibody staining, 2 μL each of Anti-CD3e, Anti-CD4, Anti-CD8, Anti-CD44, Anti-CD62L, Anti-CD45, Anti-B220+, Anti-CD49b, Anti-CD11b, and Anti-Ly6G+ were added and mixed thoroughly with the cells. The labeled cell suspension was incubated at room temperature for 30 min in the dark. Finally, the cells were resuspended in 300 μL of staining buffer, filtered through a sterile filter cloth to prevent clogging of the sample needle, and analyzed by flow cytometry using a FACS Aria II (BD Biosciences, San Jose, CA, USA).

### 2.9. Biochemical Analysis of Serum in Mice

Following orbital blood collection, whole blood was allowed to clot at room temperature for 30 min and then centrifuged at 3000 rpm for 10 min at 4 °C to obtain serum. Serum samples were added according to the manufacturer’s instructions for each assay, and a fully automated biochemical analyzer (XR220 Plus, Guangdong Zhongshan Xinrui Medical Equipment Technology Co., Ltd., Zhongshan, China) was used to quantify the concentrations of total protein (TP), albumin (ALB), alanine aminotransferase (ALT), aspartate aminotransferase (AST), alkaline phosphatase (ALP), creatine kinase (CK), lactate dehydrogenase (LDH), total cholesterol (TC), triglycerides (TG), high-density lipoprotein cholesterol (HDL-C), and low-density lipoprotein cholesterol (LDL-C) in mouse serum.

### 2.10. Detection of Cytokines in the Serum of Mice with Colorectal Tumors

Before the assay, reagents were equilibrated to room temperature for 30 min, and working solutions were prepared according to the manufacturer’s instructions. Serum samples were diluted 1:2 with assay buffer and used immediately. The Luminex instrument (Luminex Corporation, Minnetonka, MN, USA) was pre-heated and calibrated for 30 min prior to use. For the multiplex assay, 50 µL of vortexed magnetic bead mixture was added to each well, followed by two washes with 100 µL of wash buffer. Then, 50 µL of standards, samples, blanks, and quality controls were added to the corresponding wells. The plate was sealed and incubated on a shaker at 800 rpm for 1–2 h at room temperature. After incubation, the liquid was aspirated, and the wells were washed three times with 100 µL of wash buffer, with residual liquid carefully removed during the final wash. Next, 25 µL of diluted detection antibody was added, and the plate was incubated on a shaker at 800 rpm for 0.5–1 h at room temperature, followed by another wash. Then, 50 µL of diluted PE-streptavidin was added, and the plate was incubated in the dark on a shaker at 800 rpm for 10–30 min, followed by a final wash. Finally, 100–150 µL of assay buffer was added, and the plate was shaken at 800 rpm for 0.5–2 min before being analyzed, with data analysis performed using Milliplex Analyst software (Version 5.1).

### 2.11. Statistical Analysis

Statistical analyses were performed using SPSS 25.0 software. Data were expressed as mean ± SD. Given the factorial design, two-way analysis of variance (ANOVA) was used to assess main effects and interactions. Two separate models were applied: (1) factors “with or without FU” and “with or without pre-intervention” (to evaluate the effects of FU and pre-intervention), (2) factors “DP dose” (0, 0.33, 0.67, 1.33 g/kg) and “with or without pre-intervention” (to evaluate dose and timing effects). For repeated measurements (body weight, tumor volume), mixed effect models were used. Bonferroni adjustment was applied for multiple comparisons within each family of outcomes. A corrected *p* < 0.05 was considered statistically significant. Post hoc comparisons were performed using Bonferroni adjustment. No primary outcome or sample size was pre-specified. Therefore, the study was preliminary (as stated in the title and abstract). The cage was not used as the experimental unit. Graphs were generated using GraphPad Prism 8.0.2, and flow cytometry data were analyzed using FlowJo V10 software.

## 3. Results and Discussion

### 3.1. Effects of DP on Body Weight of Mice with Colorectal Tumor

**Figure 2:**
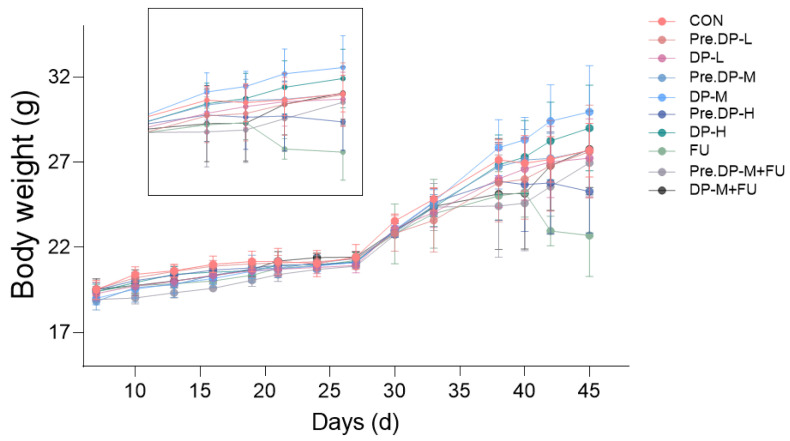
Body weight of mice with colorectal tumor in each group (*n* = 8). Data are expressed as mean ± SD.

By observing the condition of the tumor-bearing mice, we found that their fur was dry, brittle, and lackluster, and they appeared lethargic. In contrast, the mice in the treatment group had shiny fur, maintained normal water and food intake, and appeared energetic. However, these observations reflected overall trends and were inherently subjective. The analysis of mixed effect model showed that the main effect of time (*p* < 0.0001), the main effect of treatment group (*p* = 0.0407) and the interaction between time and group (*p* < 0.0001) were all significant, indicating that there were significant differences in the trajectory of mice’s weight change with time in different treatment groups. The variation between individuals (SD = 0.6146) was less than the residual variation (SD = 1.080), which indicated that the model fitting is reasonable. These findings indicated that the body weight trajectories over time differed significantly among treatment groups, and the patterns of weight change varied markedly between groups. As shown in Table 3 and Figure 2, during the early phase of the feeding period, the overall body weight trends of the mice were generally similar, with a slow upward trend. By the late feeding period, however, the differences in trends became more pronounced. This was likely due to rapid tumor progression causing cachexia in some mice within the treatment groups. On day 21, all mice were injected with CT26 cells. Seven days later (d 27), a rapid increase in body weight was observed, possibly resulting from the rapid growth of tumor tissue, which stimulated food intake and accelerated weight gain. From Table 3, it can be seen that, during the early phase (before d 30), the weight gain trends in the low-, medium- and high-dose pre-intervention groups were all superior to those in the direct intervention groups receiving the same doses, indicating that DP pre-treatment in the early stages of tumor development could significantly increase mouse body weight by improving nutritional metabolism and the mice’s physical condition. On day 42, body weight was significantly lower in the FU group compared with the CON group (*p* = 0.046), as well as compared to the DP-M group (*p* = 0.0031) and the DP-H group (*p* = 0.0172). In addition, we observed a significant and abrupt 8.98% decline in body weight in mice in the FU group on day 42 compared to day 40. This decrease exceeded the diagnostic standard for cachexia (involuntary weight loss of more than 5% within the past 6 months), and we speculated that this may be a typical manifestation of the combined effects of chemotherapy-induced side effects (such as adverse gastrointestinal reactions) and tumor-associated cachexia [19,20]. Notably, the combined intervention with DP mitigated the weight loss in the FU group. These results indicated that DP could reduce chemotherapy toxicity and maintain body weight homeostasis, thereby providing a solid foundation for the body’s tolerance during chemotherapy.

### 3.2. Effects of DP on Tumor Volume of Mice with Colorectal Tumor

To determine whether DP and FU could inhibit tumor growth, all mice were injected with CT26 cells on day 21. The volume of subcutaneous tumors was measured every 2–3 days. As shown in Table 4, 7 days after injection of CT26 cells (d 28) the tumor volume was approximately 30 mm^3^. Of note, the tumor volumes in the pre-intervention low-dose, medium-dose, and high-dose groups were all smaller than those in the direct intervention groups receiving the same doses. Table 4 revealed that, during the early intervention phase, tumor volumes in all groups showed a slow growth trend. It was found that, compared with the growth rate of tumors, the difference in tumor load between treatments was very small (*p* = 0.999). After day 33, cancer cells proliferated rapidly, and tumor volumes continued to expand, with all groups exhibiting rapid growth. The FU group exhibited relatively rapid growth in the early phase, but the growth rate slowed in the later phase (days 35–42). This trend may be related to the onset time and mechanism of action of FU. As a cell-cycle-specific drug, FU primarily blocks DNA synthesis by inhibiting thymidylate synthase and interferes with RNA function by incorporating itself into RNA as a pseudometabolite. Liu et al. [21] demonstrated that FU inhibits the proliferation of human fibrosarcoma HT-1080 cells in a time- and concentration-dependent manner, which was consistent with the gradual emergence of anti-tumor effects in the FU group during the later stages of this study. However, the analysis of the mixed effect model showed that the main effect of tumor volume over time was significant (*p* < 0.0001), while the main effect in grouping (*p* = 0.999) and the interaction between time and grouping (*p* > 0.9999) were not significant, indicating that there was no significant difference in tumor volume among the treatment groups, and the growth trend was consistent. It should also be noted that the CT26 subcutaneous model used in this study primarily reflects general solid tumor growth rather than the specific features of primary colorectal cancer (e.g., gut microbiota involvement or mucosal immunity), which would require models such as APC ^Min/+^ mice. Therefore, the following discussion focuses on solid tumor biology in general. Although certain amino acids have been reported to theoretically support tumor proliferation under specific conditions, emerging evidence from recent systematic reviews and meta-analyses indicates that well-balanced protein supplementation does not promote tumor growth in the context of chemotherapy and may instead improve clinical outcomes [22,23,24,25]. In this study, DP did not increase tumor volume compared with the CON group, further supporting the safety of this nutritional intervention.

### 3.3. Effects of DP on Tumor Inhibition Rate of CT26 Tumor-Bearing Mice

As can be seen from Table 5, compared with the CON group, the tumor inhibition rates of the low-, medium- and high-dose pre-intervention groups were 7.43%, 15.41% and 14.3%, respectively, which were higher than those of the direct intervention group. Therefore, it is known that the pre-intervention group experienced a better tumor suppression effect than the intervention group. At the same time, the tumor inhibition rates of low-, medium- and high-DP intervention groups were 6.05%, 6.78% and 3.63%, respectively. The results showed that the medium-dose group experienced a better effect. The tumor inhibition rate of the FU group was the highest, which was 39.27%. There was no significant difference between the Pre.DP-M+FU group and DP-M+FU group, the values of which were 29.49% and 28.69% respectively. Because of the individual differences of mice, the tumor inhibition rate of each treatment group increased, but there was no significant difference among the groups (*p* > 0.05).

### 3.4. Effects of DP on Organ Index and Colorectal Lengths of CT26 Tumor-Bearing Mice

**Figure 3:**
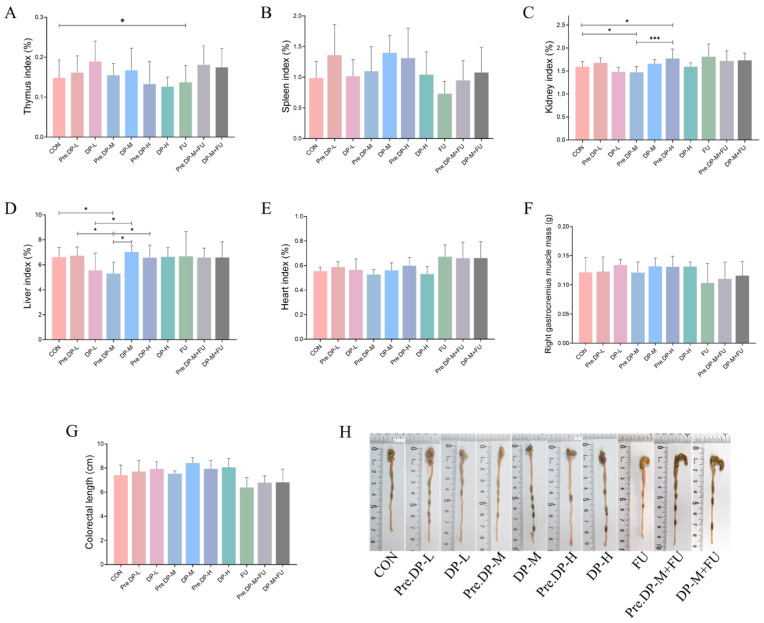
Organ indexes and colorectal lengths of CT26 tumor-bearing mice in each group (*n* = 8). (**A**) Thymus index. (**B**) Spleen index. (**C**) Kidney index. (**D**) Liver index. (**E**) Heart index. (**F**) Right gastrocnemius muscle mass. (**G**) Changes in colorectal length. (**H**) Representative images. Values are mean ± SD. Asterisks indicate statistically significant differences (* *p* < 0.05, *** *p* < 0.001, two-way ANOVA followed by Bonferroni test adjustment).

As we all know, from the point of view of morphological atrophy and functional decline, the immune system, particularly the thymus, is the first to show obvious signs of aging in mice [26]. As a key lymphoid organ in the body, the thymus is central to the development, differentiation, and maturation of T lymphocytes. A study confirmed that tumors cause progressive atrophy of the thymus in tumor-bearing mice, resulting in a significant decrease in the thymic index [27,28]. As shown in Figure 3A, the thymus index in the low- and medium-dose DP groups was higher than that in the CON group. Although the index of high-dose group was slightly lower than that of the CON group, there were no significant differences among the groups (*p* > 0.999). Furthermore, FU exhibited potent cytotoxicity, directly and severely damaging rapidly proliferating thymocytes and exacerbating thymic atrophy, resulting in a significantly lower thymus index compared to the CON group (*p* = 0.0474). In contrast, in the two combined treatment groups the thymus index increased by 24.14% and 21.48%, respectively, indicating that DP could, to some extent, alleviate FU-induced thymic atrophy.

The spleen is the body’s largest immune organ, and its importance is self-evident. As a key tissue, it plays a vital role in the study of tumor immunology [29]. Our findings in Figure 3B showed that, compared with the CON group (1 ± 0.27%), the spleen index increased in all DP-treated groups (between 1% and 1.4%). These results were consistent with our team’s previous findings in a study of mice that underwent allogeneic hematopoietic stem cell transplantation [15]. The FU group showed a lower value compared to the CON group, at only 0.73 ± 0.2%, and those of the Pre.DP-M+FU and DP-M+FU groups increased to 0.95 ± 0.32% and 1.08 ± 0.41%, respectively. The analysis revealed a significant main effect of FU treatment (*p* = 0.0251) and pre-intervention (*p* = 0.0172), while the interaction between the two factors was not significant (*p* = 0.7929). These results indicated that FU chemotherapy independently reduced the spleen index, and the pre-intervention mode (compared with direct intervention) independently increased the spleen index, with no synergistic or antagonistic interaction between them. Therefore, we speculate that FU may cause damage to immune organs, leading to a decrease in the spleen index. Meanwhile, the pre-intervention use of DP may, to some extent, alleviate tumor-induced immunosuppression and the toxic side effects of chemotherapeutic agents, as reflected by a higher spleen index.

As one of the organs responsible for metabolism and excretion, the kidneys primarily reflect the direct toxicity of chemotherapy drugs as well as the protective effects of DP nutritional support. As shown in Figure 3C, the kidney index of the CON group was 1.59± 0.11% and, with the exception of Pre.DP-H (1.77%), it ranged from 1.47 to 1.67%, remaining largely consistent and showing only slight variations. The combination therapy groups showed a kidney index of 1.72% and 1.73%, slightly lower than that of the FU group. The two-way ANOVA revealed a significant interaction between pre-intervention timing and DP dose (*p* = 0.0003), while the main effect of dose was marginally non-significant (*p* = 0.0589) and the main effect of pre-intervention was not significant (*p* = 0.1581). This indicates that the effect of pre-intervention on kidney index depends on the DP dose. Therefore, it may be inferred that DP provides sufficient EAAs, which may improve overall metabolism and protein synthesis in the body, support kidney tissue health, and exhibit no toxicity. In this study, FU treatment had a significant main effect (*p* = 0.0016). Therefore, FU significantly increased renal index, indicating nephrotoxicity. Since FU and its metabolites are excreted by the kidneys, they may cause injury to the kidney tubules and interstitial nephritis in mice, leading to renal edema and, consequently, an elevated kidney index [30,31]. Although the combination therapy slightly reduced the kidney index, this reduction did not reach statistical synergy significance.

The liver is one of the body’s essential core organs, primarily responsible for important functions such as metabolic detoxification, immune defense, and protein synthesis. The analysis revealed a significant interaction between pre-intervention timing and DP dose (*p* = 0.0005), whereas the main effects of pre-intervention (*p* = 0.493) and dose (*p* = 0.2717) were not significant. This indicates that the effect of pre-intervention on liver index depends on the DP dose. Specifically, at the medium dose, the liver index of the pre-intervention group (Pre.DP-M, 5.2%) was significantly lower than that of the direct intervention group (DP-M, 7.1%) (indicated by * in Figure 3D). At low and high doses, no significant differences were observed between pre-intervention and direct intervention groups. No other comparisons reached statistical significance. Collectively, the effect of DP intervention on liver index is dose-dependent, with the pre-intervention at a medium dose significantly reducing the liver index. Generally speaking, dietary protein intake provides the liver with the EAAs needed for protein synthesis, which may help improve hepatic steatosis caused by tumor cachexia or malnutrition, thereby restoring liver weight from the “pathological compensation” state observed in the CON group to a healthy level [32].

The heart is the central driving force of the circulatory system. Through rhythmic contraction and relaxation, it supplies essential blood and oxygen to all tissues and organs throughout the body while removing waste products and carbon dioxide, thereby sustaining the body’s vital functions. Experts and scholars have found that FU may cause cardiac toxicity, including adverse reactions such as angina, myocardial infarction/ischemia, arrhythmia, and heart failure [33]. Tumors or drugs may trigger compensatory or pathological responses characterized by increased cardiac workload and inflammation. Compared with the CON group (Figure 3E), the DP treatment group showed no significant changes (*p* > 0.05), exhibiting only slight fluctuations. However, the two-way ANOVA revealed a significant main effect of FU treatment (*p* = 0.0001), whereas the main effect of pre-intervention (*p* = 0.8032) and the interaction (*p* = 0.8769) were not significant. This indicated that FU alone significantly increased the heart index, suggesting possible myocardial injury or compensatory changes. DP intervention (with or without pre-intervention) did not significantly affect the heart index nor attenuate the FU-induced increase.

Figure 3F shows that, compared with the CON group, the DP treatment groups all exhibited a slight increase in gastrocnemius muscle mass, resulting in a greater or lesser increase in the right gastrocnemius muscle content in mice. Among these, the DP-L group had the highest value, followed by the DP-M group. Conversely, the FU group had the lowest gastrocnemius mass, at only 0.1 ± 0.03 g, while the combined DP and FU intervention group showed a slight increase in gastrocnemius mass compared to the FU group, further indicating the beneficial effect of DP on gastrocnemius mass. However, to statistically evaluate the effects of FU and pre-intervention, a factorial analysis was performed. The analysis revealed a significant main effect of FU treatment (*p* = 0.0298), while the main effect of pre-intervention (*p* = 0.5484) and the interaction (*p* = 0.7801) were not significant. These results indicate that FU significantly reduced gastrocnemius mass, consistent with its cachexia-inducing effect (as also evidenced by body weight loss in Table 3 and Figure 2). Although DP combined with FU showed numerical improvements, the lack of a significant interaction or pre-intervention effect suggests that DP did not statistically mitigate FU-induced muscle loss in this study.

In colorectal tumors, FU may reduce tumor volume by inhibiting the proliferation of tumor cells, potentially shortening the length of the colon. In addition, FU may be toxic to normal intestinal cells, causing inflammation, ulcers, or necrosis [34]. The length of the colon and rectum is closely associated with intestinal barrier function and the state of intestinal inflammation. A longer colon and rectum are typically associated with a healthier intestinal microenvironment and stronger barrier function. However, most studies have focused on inflammatory bowel diseases such as colitis [35,36,37]. Furthermore, the team’s preliminary research found that DP intervention could significantly improve colon length in mice with DSS-induced colitis. Although colon length has been widely validated as a marker in inflammatory bowel disease research, using it as an indicator to evaluate the comprehensive effects of interventions for colorectal cancer remains a relatively new research perspective. Based on this conclusion, this study used ImageJ (Version 1.53k) software to measure the length of the mouse colon and rectum. The results shown in Figure 3G,H revealed that, compared to the CON group (7.42 ± 0.83 cm), those of the DP-treated groups were all longer. Specifically, the low-, medium-, and high-dose DP intervention groups exhibited longer colon and rectum lengths than the pre-intervention groups receiving the same doses. Among these, the DP-M group had the longest length at 8.42 ± 0.44 cm, followed by the DP-H group at 8.07 ± 0.73 cm. Mice in treatment groups receiving the chemotherapeutic drug FU all exhibited shorter rectal lengths, with the FU-only group having the shortest length at 6.39 ± 0.82 cm. In the combined treatment groups, DP moderately prolonged the length, increasing it by 6.43% and 6.79%, respectively. The factorial analysis revealed a strong main effect of FU treatment (*p* = 0.0002), while the effect of pre-intervention (*p* = 0.0918) and the interaction (*p* = 0.3931) were not significant. Thus, FU significantly shortened colorectum length, whereas DP (with or without pre-intervention) did not statistically mitigate this shortening.

### 3.5. Effects of DP on Blood Routine Analysis of CT26 Tumor-Bearing Mice

**Figure 4:**
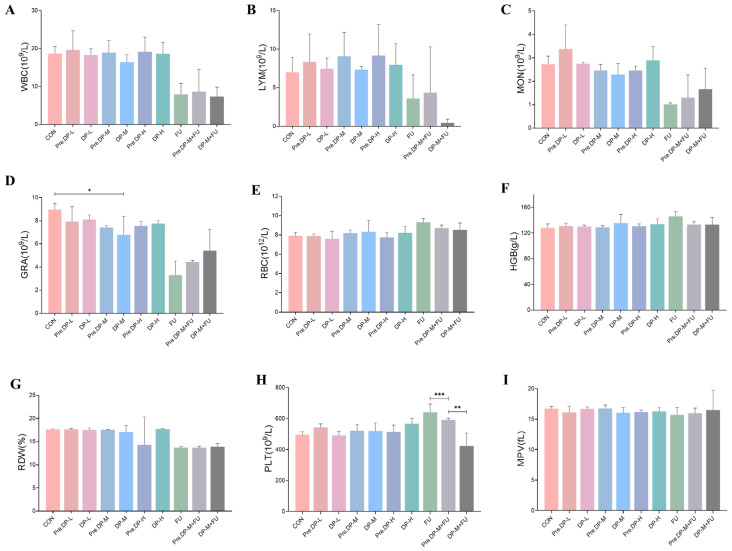
Quantitative results of whole blood of CT26 tumor-bearing mice in each group (*n* = 3). (**A**) White blood cells. (**B**) Lymphocytes. (**C**) Monocytes. (**D**) Granulocytes. (**E**) Red blood cell count. (**F**) Hemoglobin concentration. (**G**) Red blood cell distribution width. (**H**) Platelet count. (**I**) Mean platelet volume. Values are mean ± SD. Asterisks indicate statistically significant differences (* *p* < 0.05, ** *p* < 0.01, *** *p* < 0.001, two-way ANOVA followed by Bonferroni test adjustment).

White blood cell (WBC) subsets consist of lymphocytes (LYM), monocytes (MON), and granulocytes (GRA). As shown in Figure 4A, factorial analysis revealed a strong main effect of FU (*p* < 0.0001), while the pre-intervention (*p* = 0.6029) and interaction (*p* = 0.8926) were not significant. Compared with the CON group, the DP-treated group exhibited slight variations, with no significant differences observed between the two groups (*p* > 0.999). However, WBC levels in the whole blood of mice in the FU group decreased to 7.93 ± 2.91 × 10^9^/L, while those of the Pre.DP-M+FU and DP-M+FU groups were 8.63 ± 5.88 × 10^9^/L and 7.4 ± 2.46 × 10^9^/L, respectively. The absolute WBC count in whole blood of mice in the DP treatment group was higher after pre-intervention compared to the group receiving the same dose of intervention. Furthermore, the results showed that the WBC level in whole blood increased by 8.87% compared to the FU group, partially alleviating the leukopenia caused by FU.

Generally, higher LYM levels indicate a better immune status, whereas chemotherapy drugs typically cause bone marrow suppression, leading to a significant decrease in LYM cells. In Figure 4B, when compared to the CON group, LYM counts were universally elevated in all DP treatment groups, but there was no significant difference (*p* > 0.999). Consistent with the previously mentioned WBC results, the pre-intervention group exhibited higher LYM counts than the group receiving the same dose of intervention, suggesting that preventive intervention may provide better protective effects. In contrast, factorial analysis revealed a significant main effect of FU (*p* = 0.0012), while pre-intervention (*p* = 0.4163) and interaction (*p* = 0.5151) were not significant. The FU group showed markedly lower LYM counts (3.62 ± 3.04 × 10^9^/L) compared with the CON group, confirming FU-induced lymphopenia.

Figure 4C shows that, compared with the CON group, MON counts in the various DP dose groups exhibited slight increases and decreases. Notably, MON levels were higher in the Pre.DP-L and DP-H groups, suggesting that DP may possess a better ability to maintain immune function. However, factorial analysis revealed a significant main effect of FU (*p* = 0.0014), while the pre-intervention (*p* = 0.9457) and interaction (*p* = 0.3155) were not significant. The FU group had the lowest MON count, at 1.01 ± 0.062 × 10^9^/L, indicating a significant and potent inhibitory effect of the chemotherapy drug on myeloid immune cells. Meanwhile, MON counts in the Pre.DP-M+FU and DP-M+FU groups recovered to 1.3 ± 0.96 × 10^9^/L and 1.67 ± 0.89 × 10^9^/L, respectively. The results indicated that, compared with the FU group, the combination with DP partially mitigated the reduction in MON levels caused by FU, but there was no significant difference (*p* = 0.609).

GRA are a kind of white blood cell with special particles, mainly including neutrophils, eosinophils and basophils, which participate in the immune and inflammatory reactions of the body, mainly neutrophils in mice. Studies have shown that the tumor-bearing state in colorectal cancer alone triggers tumor-associated inflammation in mice, and when tumor metastasis occurs, it often coincides with a mild elevation in neutrophil counts [38,39]. As shown in Figure 4D, the CON group had the highest GRA levels, which may reflect a systemic inflammatory response induced by tumor burden. GRA levels decreased in all treatment groups, with the FU group showing the lowest levels, while the combination therapy group exhibited higher levels than the FU group. The factorial analyses were performed separately for groups without FU and with FU. In the absence of FU, DP dose had a significant main effect (*p* = 0.0073), while the pre-intervention (*p* = 0.8501) and interaction (*p* = 0.786) were not significant, indicating that DP dose independently influenced GRA levels. In the presence of FU, a two-way ANOVA (factors: FU, pre-intervention) revealed a strong main effect of FU (*p* < 0.0001) and a significant interaction (*p* = 0.0204), whereas pre-intervention alone was not significant (*p* = 0.9407). The literature indicated that neutropenia is the most common adverse reaction associated with FU monotherapy or in combination with chemotherapy in clinical practice, and it serves as the primary basis for dose adjustment and delayed administration during chemotherapy [40]. Our experiment showed that DP could effectively alleviate this side effect, which is one of the important advantages of the joint intervention program.

Building on the analysis of white blood cell changes, this study further examined red-blood-cell-related parameters, including red blood cell (RBC) count, hemoglobin concentration (HGB), and red blood cell distribution width (RDW), to comprehensively assess whether DP and FU affect anemia in tumor-bearing mice. For RBC count (Figure 4E), factorial analysis revealed a significant main effect of FU (*p* = 0.0297), while the pre-intervention and interaction were not significant. The FU group had the highest RBC levels at 9.33 ± 0.41 × 10^12^/L, which may be related to reduced tumor burden and temporary relief of bone marrow hematopoietic suppression following chemotherapy [41]. The combined intervention group maintained the stimulatory effect on RBC production, with the pre-intervention using DP achieving better results.

HGB, as a key indicator of red blood cell oxygen-carrying capacity, is the gold standard for diagnosing tumor-associated anemia [42]. From Figure 4F, there were no significant differences among groups (*p* > 0.05). The FU group had the highest HGB level, followed by the DP-M group. No evidence of anemia or impaired oxygen-carrying capacity in mice due to chemotherapy-induced HGB synthesis impairment was observed.

RDW, an indicator of red blood cell morphological heterogeneity, exhibited significant differences across groups, revealing the impact of chemotherapy on the microscopic morphology of red blood cells. A very strong main effect of FU was found (*p* < 0.0001), whereas other effects were not significant, suggesting that the morphological characteristics of red blood cells were primarily dominated by FU. Specifically, the RDW level (Figure 4G) in the CON group was 17.6 ± 0.12%. Except for the Pre.DP-H group and DP-M, the RDW levels in the other DP dose groups were generally consistent with that of the CON group, ranging between 17.5% and 17.7%, with relatively small fluctuations. Among the combination treatment groups, the Pre.DP-M+FU group showed the lowest RDW value at 13.7 ± 0.3%, followed by the DP-M+FU group at 13.9 ± 0.66%. Hence, we speculated that FU significantly altered the volume distribution characteristics of whole blood red blood cells, possibly reflecting changes in bone marrow hematopoietic activity. Pre-intervention with DP appeared to appropriately influence this change, suggesting that it may act by protecting bone marrow hematopoietic function or promoting the recovery of the hematopoietic system. This supported our previous findings in mice undergoing allogeneic hematopoietic stem cell transplantation [15,43].

On the basis of previous research results of white blood cell line and red blood cell line, the changes of platelet (PLT) and mean platelet volume (MPV) were studied further. As shown in Figure 4H, the PLT levels in the CON group were lower, suggesting potentially impaired coagulation. Most DP-treated groups (except the DP-L group) exhibited a trend toward a slight increase, ranging from 514–565 × 10^9^/L, indicating that DP promotes PLT production and moderately alleviates tumor-induced suppression of bone marrow hematopoiesis. The PLT levels in mice in the medium-dose DP group were relatively stable. It is worth noting that the FU group exhibited the highest PLT levels, which may be attributed to a temporary relief of bone marrow hematopoietic suppression leading to a significant increase in platelet production. Meanwhile, factorial analysis revealed a significant interaction between pre-intervention and FU (*p* = 0.0034) and a significant main effect of pre-intervention (*p* = 0.0099), while the main effect of FU was not significant (*p* = 0.1178). Thus, PLT levels were influenced by DP pre-intervention and its combination with FU, rather than by FU alone. The combined intervention group showed different results, possibly due to the varying regulatory effects of the different interventions on the bone marrow hematopoietic microenvironment at different time points. Preventive DP intervention not only provided an early supply of amino acids necessary for hematopoiesis, protected hematopoietic stem cells in the bone marrow, and mitigated the direct damage caused by subsequent chemotherapy but also built up nutritional reserves to rapidly support platelet production after chemotherapy and prevent a sharp decline. Conversely, the combined metabolic stress from chemotherapy and DP would simultaneously impact the bone marrow microenvironment, exacerbating damage to hematopoietic stem cells and leading to insufficient PLT production.

MPV reflects platelet size, with larger platelets typically indicating younger, more active platelets and reflecting platelet maturity. The MPV levels in the blood of tumor-bearing mice across all groups were highly similar, ranging from 15.7 to 16.73 fL (Figure 4I), showing a slight upward or downward trend. The Pre.DP-M group had the highest value at 16.73 ± 0.59 fL, suggesting that under the moderate-dose DP pre-intervention strategy, the quality and functional activity of PLT production in the whole blood of tumor-bearing mice were superior. Overall, DP pre-intervention appeared to maintain platelet homeostasis more effectively, potentially by preserving bone marrow hematopoiesis and promoting platelet maturation. This provided a stable hematopoietic platform for subsequent combination treatments, indicating its potential as a superior comprehensive therapeutic strategy.

### 3.6. Effects of DP on Splenic Immune Cell Profiles of CT26 Tumor-Bearing Mice

#### 3.6.1. Effects of DP on T Cells in Spleen Tissue

**Figure 5:**
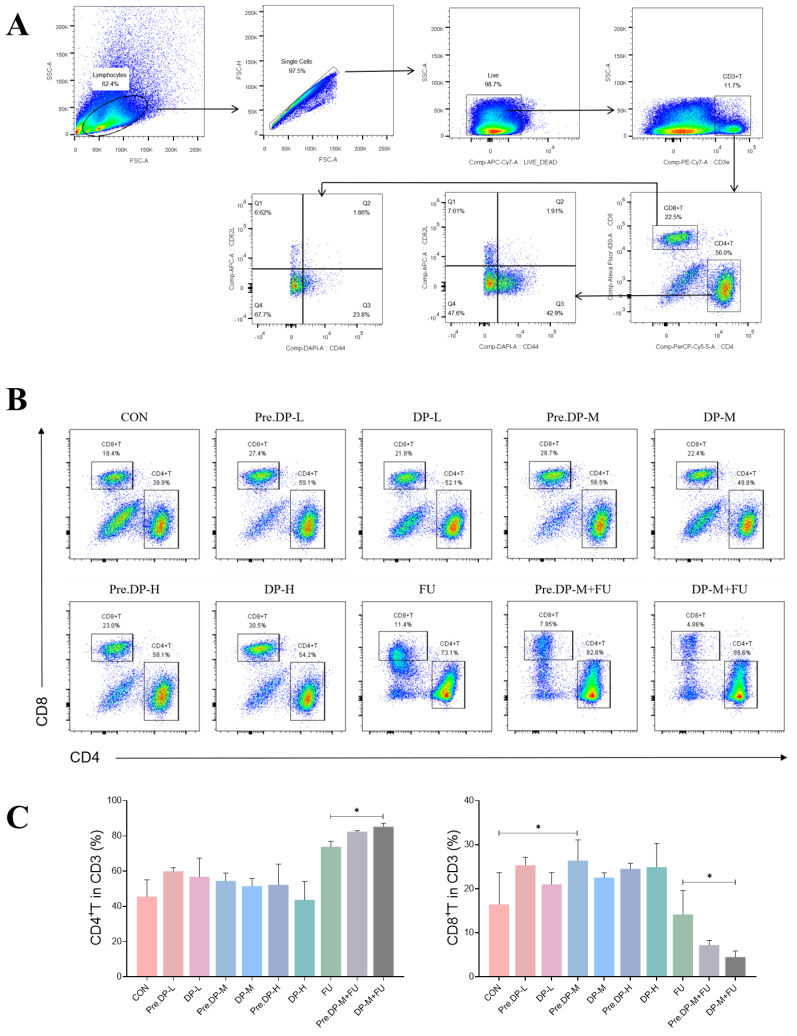
Proportion of mouse spleen T cells. (**A**) Gating strategy. (**B**) Representative flowchart. (**C**) The proportion of CD4^+^T and CD8^+^T cells in spleen tissue of mice in each group. Values are mean ± SD. Asterisks indicate statistically significant differences (* *p* < 0.05, two-way ANOVA followed by Bonferroni test adjustment) (*n* = 3).

Among T cell subsets, T helper cells (CD4^+^T) and T cytotoxic cells (CD8^+^T) play central roles in the immune system. CD4^+^T cells primarily play a regulatory role in the adaptive immune response, such as by secreting cytokines, activating macrophages, assisting B cells in producing antibodies and supporting CD8^+^T cell responses. These functions help coordinate the adaptive immune response, facilitate the recruitment and activation of more effector cells, and thereby establish a more robust immune surveillance network [44,45]. CD8^+^T, on the other hand, function as cytotoxic T lymphocytes by directly killing virus-infected cells and tumor cells through the release of effector molecules such as perforin and granzyme, performing an immune surveillance role [46,47]. Through mutual regulation and synergistic action, these two cell types jointly maintain the body’s immune balance [48]. However, during tumor progression, the chronic inflammatory microenvironment often leads to an imbalance in the proportions of T lymphocyte subsets and functional dysregulation [49]. Therefore, in this study, flow cytometry was used to assess the changes in T cell populations in spleen tissues, and gating analysis was performed using Flowjo V10 software (Figure 5A). As shown in Figure 5B,C, the proportion of CD4^+^T cells in spleen tissues of the CON group was 45.6 ± 9.44%. Except for the DP-H group, the CD4^+^T cell percentages in the DP treatment groups were increased, which was consistent with the findings of our previous study [17]. The pre-intervention strategy outperformed the corresponding intervention groups at all dose levels, suggesting that early nutritional intervention offers unique advantages in maintaining CD4^+^T cell homeostasis and may lay the foundation for subsequent anti-tumor immunity by regulating the immune axis prior to tumor development. Importantly, factorial analysis revealed a strong main effect of FU (*p* < 0.0001) and a significant main effect of pre-intervention (*p* = 0.0130), while the interaction was not significant (*p* = 0.5318). The CD4^+^T cell proportion in the FU group abnormally increased to 73.8–85.2%, significantly higher than in other groups. This phenomenon may be related to antigen-specific T cell responses induced by the immunogenic cell death effects of FU. Levels were higher in the combination group than in the FU group, but because the interaction was not significant, the numerical value was higher than that of the FU group, which could not represent the statistical synergistic effect. On the contrary, FU and pre-intervention independently affected CD4^+^T cell response.

The results in Figure 5C showed that, in the CD8^+^ T subpopulation analysis, the CON group accounted for 16.45 ± 7.23%, indicating impaired cytotoxic T cell function in the tumor microenvironment and significant interindividual variability. Two-way ANOVA (factors: pre-intervention, DP dose) revealed a significant main effect of DP dose (*p* = 0.0207), while pre-intervention (*p* = 0.3155) and interaction (*p* = 0.7279) were not significant. The DP group showed an increase compared to the CON group, with Pre.DP-L (25.33 ± 1.81%) and Pre.DP-M (26.4 ± 4.69%) exhibiting slightly greater increases. This indicated that DP, when administered at an appropriate dose, was effective in promoting the proliferation and infiltration of CD8^+^T cells and enhancing their direct tumor-killing capacity. In groups receiving FU, another two-way ANOVA (factors: FU, pre-intervention) showed a strong main effect of FU (*p* < 0.0001) and a significant interaction (*p* = 0.0077). Yet the CD8^+^T cell proportions in the FU group, Pre.DP-M+FU group, and DP-M+FU group decreased to 14.2 ± 5.38%, 7.21 ± 1.08%, and 4.49 ± 1.39%, respectively. This phenomenon revealed FU’s potential immunotoxicity, where high-intensity tumor killing may also indiscriminately suppress or deplete CD8^+^T cells. In the combination intervention groups, the decrease in CD8^+^T cells may be due to a relative decline caused by the extreme expansion of CD4^+^T cells (85.2%) or may reflect a redistribution process of effector CD8^+^T cells.

The above results showed that DP intervention affected the proportional distribution of spleen T lymphocytes. In order to further clarify the functional state and differentiation stage of these T cells, we detected the expression levels of CD44 and CD62L on the surface of T cells, so as to distinguish naïve T cells (CD44^lo^CD62L^hi^), effector memory T cells (CD44^hi^CD62L^lo^, Tem) and central memory T cells (CD44^hi^CD62L^hi^, Tcm), thereby providing deeper insights into the mechanism by which DP acted in anti-tumor immunity.

**Figure 6:**
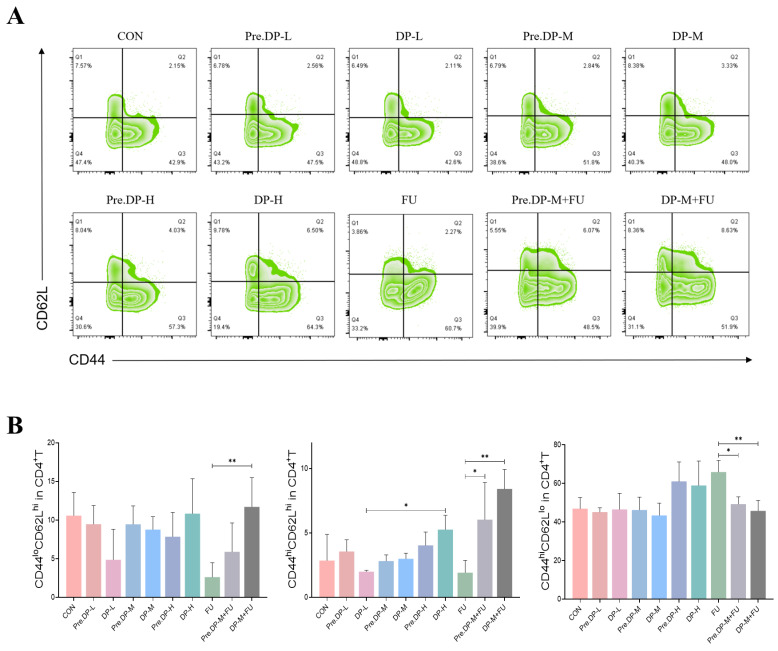
Expression of CD44 and CD62L in CD4^+^T cells. (**A**) Representative flowchart. (**B**) Naïve (CD44^lo^CD62L^hi^) CD4^+^T, central memory (CD44^hi^CD62L^hi^) CD4^+^T and effector memory (CD44^hi^CD62L^lo^) CD4^+^T cell population ratio. Values are mean ± SD. Asterisks indicate statistically significant differences (* *p* < 0.05, ** *p* < 0.01, two-way ANOVA followed by Bonferroni test adjustment) (*n* = 3).

From Figure 6, the spleen of CON mice showed a naïve CD4^+^ T cell proportion of 10.59 ± 3.02%, with CD4^+^ Tem cells at 46.92 ± 5.78% and CD4^+^ Tcm cells as low as 2.86 ± 2.04%. This pattern indicated that, under tumor burden, the body exhibited a certain effector immune response, yet lacked sufficient immune memory formation. It also implied that the naïve T cell pool may be depleted through continuous activation. DP intervention, particularly at high doses, exerted pronounced immunomodulatory effects. Two-way ANOVA confirmed that DP dose significantly regulated both Tcm (*p* = 0.0484) and Tem (*p* = 0.015). In the DP-H group, Tcm cells increased to 5.26 ± 1.11%, while Tem cells remained elevated (58.93 ± 12.64%), and the Pre.DP-H group showed further increased effector responses (61.07 ± 10.09%). These findings revealed that DP not only promoted the differentiation of naïve T cells into effector phenotypes to boost immediate cytotoxicity but also enhanced the generation of central memory T cells. Because central memory T cells were essential for long-term immune surveillance and contributed to an extended anti-tumor memory reservoir, DP helped prevent tumor recurrence [50]. The FU group exhibited the highest Tem cell proportion (65.93 ± 5.95%) but failed to support Tcm cell formation (1.92 ± 0.95%), and naïve T cells decreased to 2.63 ± 1.86%, consistent with chemotherapy-driven immune suppression of memory formation and depletion of the naïve T cell pool. Two-way ANOVA revealed significant interaction between pre-intervention of DP and FU for naïve CD4^+^ T cells (*p* = 0.0201) and Tcm (*p* = 0.017). Significant main effects were also observed, where FU significantly affected Tcm (*p* = 0.0061) and CD4^+^ Tem cells (*p* = 0.0182), while pre-intervention significantly influenced Tcm (*p* = 0.0137) and Tem (*p* = 0.0051). In contrast, the DP-M+FU combination showed a trend effect by restoring naïve T cells to 11.72 ± 3.77% and elevating Tcm cells to 8.42 ± 1.52%. Compared with FU alone, the combination markedly improved immune memory and replenishes the naïve T cell pool. This result indicated that DP counteracts chemotherapy-induced immune exhaustion, protected naïve T cells and promoted memory differentiation. The Pre.DP-M+FU group, showing 6.04 ± 2.88% central memory T cells, further confirmed its superiority over FU monotherapy. Overall, this rebalancing of effector–memory immunity highlighted DP’s dual function in safeguarding immune reserves and inducing memory T cells. DP combined with FU does not just heighten immediate anti-tumor efficacy. Crucially, it established robust immune surveillance and may result in superior long-term outcomes for colorectal tumor therapy, offering a new strategy that strengthens chemotherapy’s tumor suppression with supportive immune protection.

**Figure 7:**
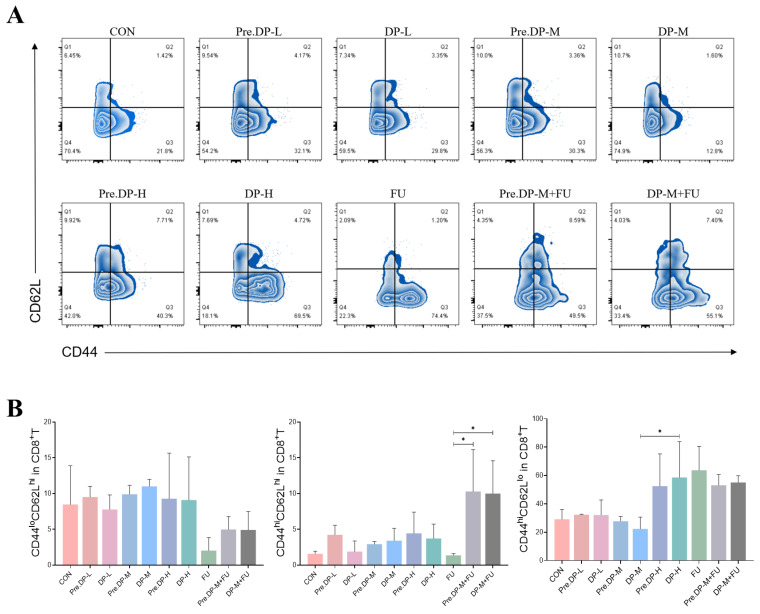
Expression of CD44 and CD62L in CD8^+^T cells. (**A**) Representative flowchart. (**B**) Naïve (CD44^lo^CD62L^hi^) CD8^+^T, central memory (CD44^hi^CD62L^hi^) CD8^+^T and effect memory (CD44^hi^CD62L^lo^) CD8^+^T cell population ratio. Values are mean ± SD. Asterisks indicate statistically significant differences (* *p* < 0.05, two-way ANOVA followed by Bonferroni test adjustment) (*n* = 3).

Flow cytometry analysis (Figure 7B) showed that, in the CON group, splenic naïve CD8^+^T cells accounted for 8.47 ± 5.41%, CD8^+^Tem cells for 29.1 ± 6.93%, and CD8^+^Tcm cells for only 1.59 ± 0.36%. These data indicated that the tumor microenvironment drove the depletion of cytotoxic T lymphocyte precursors and impaired immune memory formation. Factorial analyses were performed separately for groups without FU (dose and pre-intervention effects) and with FU (FU and pre-intervention effects). In the absence of FU, DP dose had a significant main effect on CD8^+^ Tem cells (*p* = 0.0053), while pre-intervention and interaction were not significant. Regarding CD8^+^Tcm in the presence of FU, both FU (*p* = 0.0186) and pre-intervention (*p* = 0.0479) showed significant main effects, but the interaction was not significant (*p* = 0.0658). Only the FU main effect was significant for naïve CD8^+^T (*p* = 0.0007) and Tem (*p* < 0.0001). In particular, the Pre.DP-H group markedly increased the proportion of Tcm cells to 4.43 ± 2.98%, followed by Pre.DP-L at 4.24 ± 1.33%. DP intervention also preserved naïve CD8^+^T cells, as reflected by the higher levels in the Pre.DP-M (9.9 ± 1.26%) and DP-M (11 ± 0.98%) groups. The medium dose of DP safeguarded the immune reserve and strengthened the initial response potential against colorectal tumors. In contrast, the FU group showed the highest Tem cell proportion (63.67 ± 16.97%), but its naïve T cells dropped to 2.03 ± 1.81% and Tcm cells remained low (1.37 ± 0.23%). Although chemotherapy stimulated Tem cell expansion, it apparently depleted the naïve pool and limited immune memory, consistent with a typical side effect that may explain why chemotherapy alone often leads to immune escape and relapse in colon cancer patients. The combination groups revealed DP’s ability to modulate chemotherapy-induced changes. In DP-M+FU, Tcm cells rose to 9.99 ± 4.60%, and in Pre.DP-M+FU they reached 10.29 ± 5.85%, greatly surpassing both the FU group and the CON group. Tem cells stayed high as well, at 55.07 ± 4.75% in DP-M+FU and 53.1 ± 7.57% in Pre.DP-M+FU. Notably, the naïve T cell proportion in the combination group (4.99% and 4.92%) was improved compared with that in the FU group. This difference likely reflected DP’s support for lymphocyte homeostasis, counteracting part of the chemotherapy-induced depletion. However, the limitation of this experiment lies in the small sample size, which needs to be verified by larger-sample research in the future.

#### 3.6.2. Effects of DP on B Cells, NK Cells and Neutrophils in Spleen Tissue

**Figure 8:**
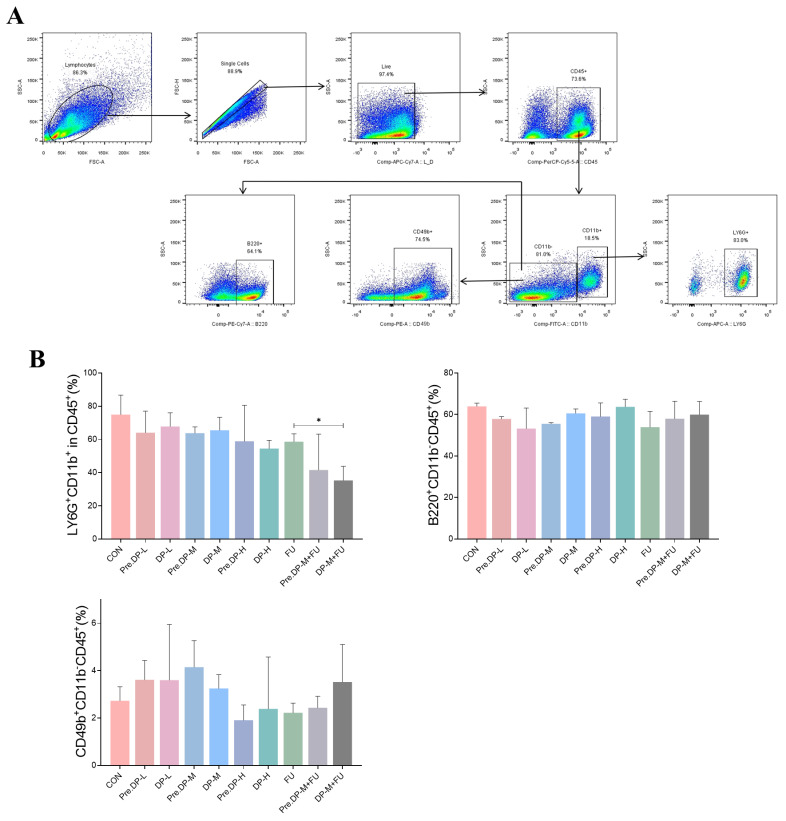
Gating strategy of flow cytometry (**A**) and the proportion of neutrophils, B cells and NK cells in spleen tissue of mice in each group (*n* = 3) (**B**). Values are mean ± SD. Asterisks indicate statistically significant differences (* *p* < 0.05, two-way ANOVA followed by Bonferroni test adjustment).

Preliminary complete blood count analyses in tumor-bearing mice showed altered leukocyte, granulocyte, and monocyte numbers. To clarify the effects of DP on immune subsets, we used flow cytometry to examine splenic neutrophils (Ly6G^+^CD11b^+^CD45^+^), B cells (B220^+^CD11b^-^CD45^+^), and natural killer (NK) cells (CD49b^+^CD11b^-^CD45^+^) in the CT26 subcutaneous tumor model. As shown in Figure 8, neutrophils dominated the CON group (74.93 ± 0.95%), suggesting tumor-associated neutrophil infiltration and a pronounced myeloid inflammatory response in the spleen, which created an immunosuppressive microenvironment. Factorial analysis revealed a significant main effect of FU (*p* = 0.0006) and a significant main effect of pre-intervention (*p* = 0.0173), while the interaction was not significant (*p* = 0.1676). Thus, both FU and pre-intervention independently reduced splenic neutrophil levels. FU-treated groups also displayed declines of 21.71%, 44.48%, and 52.89% relative to the CON group, consistent with the granulocyte findings from blood tests and reflecting the myelosuppressive effect of FU. The high-dose DP groups also exhibited the most pronounced reduction, indicating effective control of tumor-induced neutrophil overactivation, possibly by attenuating inflammatory signaling in the microenvironment and reducing recruitment of pro-tumor neutrophils. It is worth noting that, when DP was combined with FU, splenic neutrophils decreased further whereas circulating granulocytes rebounded, exemplifying a classic immunological cell redistribution. We therefore infer that DP promoted the mobilization of neutrophils from splenic stores to peripheral blood, compensating for the chemotherapy-induced drop in circulating neutrophils and providing immune protection during FU therapy. DP pre-intervention likely improved nutritional status, expanded bone marrow and splenic reserves, and slightly attenuated mobilization efficiency.

B cells contribute to anti-tumor immunity through antigen presentation, antibody production, and cytokine secretion, and maintaining their proportion is crucial for long-term immune surveillance [51]. In the analysis of B cell subsets (Figure 8B), the proportion of B cells in spleen tissue in the CON group was 63.9 ± 1.61%, reflecting a compensatory distribution under tumor burden. Two-way ANOVA revealed a significant main effect of DP dose (*p* = 0.0284). Thus, DP dose independently influenced B cell proportion. Within the pre-intervention DP group, the proportion of B cells exhibited a trend of initially decreasing and then increasing with dose. Whereas in the DP intervention group, the proportion of B cells showed a certain dose-dependent trend, lower than that in the CON group. Based on these results, we speculated that low and medium doses may reduce B cell activation by suppressing inflammatory responses, while high doses may activate adaptive immune responses. Additionally, the B cell proportion in the FU group was 53.9 ± 7.6%, suggesting that FU may exert a certain degree of toxicity or inhibitory effect on the lymphocyte pool, leading to impaired humoral immune potential. The Pre.DP-M+FU and DP-M+FU groups showed increases of 7.54% and 11.26%, respectively. However, there was no significant interaction between FU and pre-intervention, so the conclusion that DP cooperates with FU to optimize the proportion of spleen B cells still needs to be confirmed by larger samples. Therefore, DP’s optimization of the splenic B cell ratio may restore the anti-tumor immune response by balancing the dual functions of B cells, anti-tumor activity and immunosuppression.

As key effector cells of the innate immune system, the activity of NK cells is directly linked to the body’s ability to eliminate tumors at an early stage [52]. Because NK cells can recognize and kill tumor cells without prior antigen sensitization, they serve as the first line of anti-tumor defense. In this study (Figure 8B), the CON group displayed a low splenic NK cell proportion (2.73 ± 0.59%), consistent with the tumor-induced suppression of innate immunity. DP treatment at low and medium doses increased NK cells, with the Pre.DP-M group reaching the highest level (4.15 ± 1.12%). Although no statistically significant differences were detected by two-way ANOVA, this trend might suggest that DP pre-intervention could activate or expand the NK cell pool, possibly through cytokine regulation. Regarding combined interventions, the Pre.DP-M+FU group and the DP-M+FU group showed increases of 9.46% and 58.56%, respectively, compared to the FU group, but without statistical significance. Therefore, although the appropriate dose of DP may enhance the innate immune response, these findings should be regarded as preliminary results and need to be verified in larger-scale studies in the future.

### 3.7. Effects of DP on Serum Biochemical Analysis in CT26 Tumor-Bearing Mice

Blood biochemistry analysis plays a key role in cancer research. By analyzing soluble markers in the blood, it provides critical quantitative data for elucidating the systemic effects of tumors on mice, assessing organ function, determining prognosis, and monitoring the progress of interventions [53]. This study systematically evaluated the regulatory effects of DP intervention on tumor-bearing mice by measuring protein metabolism, lipid metabolism, liver function and heart enzyme profiles in their serum. Additionally, by correlating these findings with previous experimental results and our team’s research on mouse models of atherosclerosis [16], type 2 diabetes [17], leukemia [15] and burns [54], the effects of the medium-dose intervention were found to be relatively stable. Therefore, the medium-dose group was selected for subsequent serum biochemical analysis.

**Figure 9:**
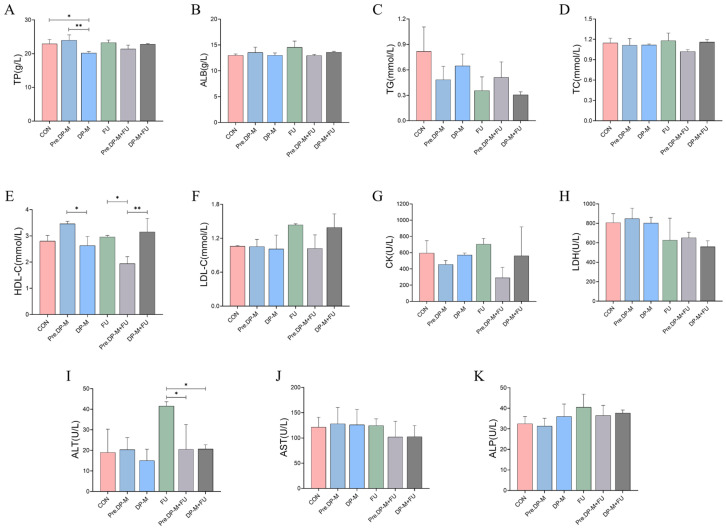
Results of serum biochemical analysis of CT26 tumor-bearing mice in each group (*n* = 3). (**A**) Total protein. (**B**) Albumin. (**C**) Triglyceride. (**D**) Total cholesterol. (**E**) High-density lipoprotein cholesterol (**F**) Low-density lipoprotein cholesterol. (**G**) Creatine kinase. (**H**) Lactate dehydrogenase. (**I**) Alanine aminotransferase. (**J**) Aspartate aminotransferase. (**K**) Alkaline phosphatase. Different letters represent significant differences between the two groups. Values are mean ± SD. Asterisks indicate statistically significant differences (* *p* < 0.05, ** *p* < 0.01, two-way ANOVA followed by Bonferroni test adjustment).

Total protein (TP) content is shown in Figure 9A. The Pre.DP-M group had the highest level (23.97 ± 1.31 g/L), while the DP-M group had the lowest (20.2 ± 0.46 g/L). Factorial analysis revealed a significant interaction between pre-intervention and FU treatment (*p* = 0.0084), whereas the main effects of FU (*p* = 0.8311) and pre-intervention (*p* = 0.0725) were not significant. This indicated that the effect of DP on serum TP depends on both the timing of intervention and the presence of FU. Specifically, pre-intervention with DP alone (Pre.DP-M) elevated TP, while direct DP intervention (DP-M) lowered TP, and these differences were modified by FU co-treatment. One possible explanation is that pre-intervention improves hepatic protein synthesis and counteracts tumor-induced catabolism, whereas direct DP intervention may transiently increase metabolic stress. FU alone did not reduce TP, possibly because chemotherapy-induced tumor shrinkage (Table 4) alleviated protein catabolism, offsetting any direct protein-suppressing effect. In combination groups, TP levels returned to near-CON values, suggesting that DP partially mitigated FU toxicity, but the significant interaction implies that the combined effect is not simply additive.

Albumin (ALB) has long been regarded as a nutritional marker for assessing an individual’s nutritional status. The progression of cancer can affect nutritional status, and ALB levels tend to decrease in patients with malignant tumors. However, in recent years, ALB has increasingly been recognized as a key indicator of systemic inflammatory response during the course of malignant tumors, rather than being associated with malnutrition [55]. It can be seen from Figure 9B that there is no significant difference in serum ALB levels among the mice in each group (*p* > 0.05). According to the analysis of the experimental results, it was found that the highest value in the FU group was 14.55 ± 1.2 g/L, which may be due to alleviating the inflammatory stress induced by the tumor and reducing the metabolic burden of the liver. The reduction of tumor consumption indirectly increased the nutritional substrate of synthetic protein in the liver, thus promoting the synthesis of ALB.

Because of their rapid proliferation, colorectal cancer cells require elevated cholesterol levels to support membrane biogenesis and other metabolic demands. Cholesterol dysregulation in these cells includes increased biosynthesis, enhanced cholesterol esterification and derivatives, and changes in the immune microenvironment. Such alterations facilitate tumor progression, including cell proliferation, migration, and invasion [56,57]. Lipoproteins carry cholesterol between intracellular and extracellular compartments and relate to intracellular accumulation. Some studies found that colorectal cancer patients exhibit decreased serum total cholesterol concentrations [58]. Investigating abnormal lipid metabolism is therefore important for individualized treatments in colorectal cancer, a topic of growing interest among clinicians [59]. We measured serum total cholesterol (TC), triglyceride (TG), high-density lipoprotein cholesterol (HDL-C), and low-density lipoprotein cholesterol (LDL-C) in tumor-bearing mice. As shown in Figure 9D, TC was lowest in the Pre.DP-M+FU group (1.02 ± 0.028 mmol/L), whereas the FU group displayed the highest value (1.18 ± 0.11 mmol/L). This trend suggested that DP pre-intervention combined with FU modestly reduced TC, although differences among groups were not statistically significant (*p* = 0.2509). For TG (Figure 9C), a significant main effect of FU was found (*p* = 0.028), while pre-intervention and interaction were not significant, indicating that FU independently reduced TG levels. Yet the CON group had the highest average (0.82 ± 0.29 mmol/L), indicating that both DP and FU reduced TG to some extent. For HDL-C (Figure 9E), a significant interaction between pre-intervention and FU was observed (*p* = 0.0009), meaning the effect of pre-intervention on HDL-C depends on FU co-treatment. Specifically, Pre.DP-M alone showed the highest HDL-C (3.46 ± 0.095 mmol/L), whereas Pre.DP-M+FU had lower HDL-C (significant differences noted in the figure), suggesting that FU blunts the HDL-C-raising effect of pre-intervention DP. In contrast, LDL-C (Figure 9F) rose by 35.38% in the FU group and 31.13% in DP-M+FU relative to the CON group, while other groups fluctuated mildly, implying that FU tended to elevate LDL-C, and FU exhibited a significant main effect (*p* = 0.0402). Overall, the Pre.DP-M group significantly increased HDL-C and lowered TG. These outcomes suggested that DP pre-intervention, with essential amino acid supplementation, regulated hepatic lipid metabolism, possibly promoting reverse cholesterol transport to tumor cells [60] and inhibiting TG synthesis. This pattern aligned with an improved lipid profile, indicating that pre-intervention was preferable. FU monotherapy reduced TG but increased LDL-C, showing a dual effect of anti-tumor activity and chemotherapy-associated toxicity (e.g., hepatocellular injury, disruption of lipid metabolism). Combined treatment (DP+FU) lowered TG synergistically, and DP pre-intervention ameliorated the FU-induced rise in LDL-C while also reducing TC, consistent with findings reported by Yuan and Yang [59]. Collectively, the Pre.DP-M+FU strategy appeared most effective.

Creatine kinase (CK) and lactate dehydrogenase (LDH) serve as biomarkers of skeletal muscle damage in pre-clinical toxicity studies [61] and are commonly measured in animal studies [62]. In this study (Figure 9G), serum CK levels in the CON group were 594.33 ± 153.44 U/L, in the Pre.DP-M group 455.5 ± 47.38 U/L, and 570.33 ± 25.17 U/L in the DP-M group. In the combined intervention group, levels decreased to 290.5 ± 127.99 U/L and 561.33 ± 355.94 U/L, respectively. Factorial analysis revealed no significant main effects and interaction. Thus, despite numerical differences, none of the treatment effects on serum CK reached statistical significance. The numerical elevation in the FU group suggests possible muscle damage, while the lower values in combination groups hint at a protective trend, but these observations require validation in larger studies. As an enzyme primarily distributed in skeletal muscle, elevated CK levels reflect the toxic effects of chemotherapy drugs on muscle tissue, which induce muscle fiber necrosis, leading to the release of intracellular CK into the bloodstream. This results in elevated serum CK levels in mice and exacerbates muscle cell damage.

LDH is a class of nicotinamide-adenine-dinucleotide-dependent kinases and one of the key enzyme systems involved in glycolysis and gluconeogenesis. It is widely distributed throughout human tissues, with the highest concentrations found in the kidneys, followed by cardiac muscle and skeletal muscle [63]. In this study (Figure 9H), compared with the CON group, serum LDH levels in mice in the DP-treated group showed a slight upward trend, which is presumed to be due to stress caused by immune activation. Factorial analysis revealed a significant main effect of FU treatment (*p* = 0.003), while the main effect of pre-intervention (*p* = 0.5829) and the interaction (P = 0.8782) were not significant. Specifically, FU-containing groups (FU alone, Pre.DP-M+FU, DP-M+FU) showed lower LDH levels (626–559 U/L) compared with non-FU groups (CON and DP-treated groups). This decrease likely reflects effective inhibition of tumor cell proliferation by FU, as rapidly dividing tumors often elevate LDH. The absence of an abnormal LDH elevation also suggests that FU was within a relatively safe range at this dosage. No synergistic effect of DP with FU was detected (non-significant interaction). The numerical differences among DP-treated groups without FU (slight upward trend) were not statistically supported.

In tumor-bearing mice, measuring serum alanine transaminase (ALT), aspartate aminotransferase (AST) and alkaline phosphatase (ALP) is a key method for assessing liver function and hepatocyte damage [64]. Studies have found that soy protein can lower serum ALT and AST levels [65,66], reduce liver damage, and promote liver cell regeneration. However, since the experimental model used was not a mouse model of colorectal cancer, these findings cannot be directly applied to mice with colorectal cancer. As a commonly used chemotherapy drug for colorectal cancer, FU exhibits significant dose-dependent hepatotoxicity alongside its anti-tumor effects [67]. Factorial analysis revealed a significant main effect of FU (*p* = 0.0271), while the main effect of pre-intervention was marginally non-significant (*p* = 0.0515) and the interaction was not significant (*p* = 0.0919). As shown in Figure 9I, compared with the CON group, ALT levels in all groups receiving DP treatment showed slight increases or decreases. In the FU group, ALT levels rose significantly to 41.5 ± 2.12 U/L, subsequently inducing drug-induced liver injury in mice. These results were consistent with those reported by Wang et al. [68] and Chen et al. [69]. It may be inferred that FU could disrupt the redox balance in the liver, induce oxidative stress, and result in hepatocyte damage. Furthermore, the results of this study found no significant differences in AST and ALP levels among the groups (*p* > 0.999), but as shown in Figure 9J, K, the combination of DP and FU intervention appropriately alleviated the hepatotoxic effects induced by FU. Therefore, from the nutritional point of view, as high-quality animal and plant protein, DP may indirectly promote the repair of hepatocytes and protect the liver by improving the nutritional status of mice, but in the tumor micro-environment, the specific role needs to be explored carefully, and the intervention opportunity of DP deserves special attention.

### 3.8. Effects of DP on Serum Cytokines in Mice with Colorectal Tumors

**Figure 10:**
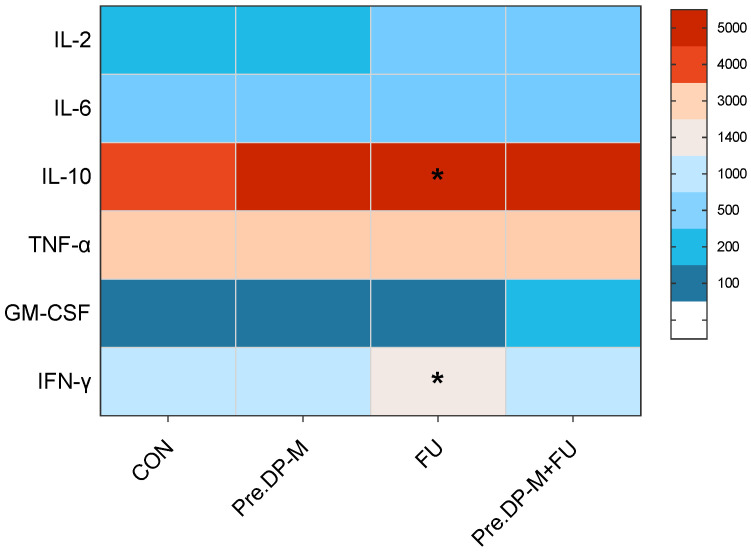
Heatmap results of serum cytokines of tumor-bearing mice in each treatment group (*n* = 3). The * in the figure indicates that there is a significant difference between the treatment group and the CON group (*p* < 0.05).

As shown in Figure 10, compared with the CON group, levels of IL-2, IL-10, GM-CSF and IFN-γ were increased, while IL-6 and TNF-α were decreased in all treatment groups. It is well known that IL-2 is an important immunoregulatory factor that promotes the proliferation and activation of T cells and NK cells, further mediating the activation of monocytes/macrophages and the production of tumor necrosis factor. Wang et al. [70] demonstrated that combined IL-2/IFN-β gene therapy is an effective anti-tumor strategy in colorectal cancer, not only inhibiting tumor growth but also preventing tumor-related mortality. Our study found that IL-2 levels were lower in the serum of mice in the CON group, possibly due to immunosuppressive cells in the tumor microenvironment inhibiting its production. In contrast, IL-2 levels were higher in the serum of tumor-bearing mice in the other treatment groups, indicating that DP, FU, and their combined intervention can significantly improve the profile of immunosuppressive cells in the tumor microenvironment, thus inhibiting tumor growth. Evidence has shown that enhanced IL-10 production directly leads to increased resistance of colon cancer cells to 5-FU [71]. This implied that, if IL-10 levels did not decrease significantly or even increased in the 5-FU monotherapy group, it may indicate the development of drug resistance. Meanwhile, in previous studies, the research team found that DP intervention could improve the gut microbiome by increasing the diversity and abundance of beneficial bacteria. It also increased the number and activity of Tregs through mechanisms such as mitochondrial pathways, thereby promoting the secretion of anti-inflammatory cytokines such as IL-10 [72]. The results of this study showed that IL-10 levels, from highest to lowest, were as follows: the FU group (4322.33 ± 890.22 pg/mL), the Pre.DP-M+FU group (4322.33 ± 200.14 pg/mL), the Pre.DP-M group (4098.33 ± 786.39 pg/mL), and the CON group (3934.33 ± 249.3 pg/mL). These findings revealed that DP not only protected the intestinal epithelial barrier and increased IL-10 levels but also reduced tumor resistance to FU. The decreased expression of GM-CSF in the tumor microenvironment may be closely related to the local immunosuppression dominated by immunosuppressed cells (such as macrophages and dendritic cells) [73]. In particular, serum GM-CSF levels were elevated in mice in the Pre.DP-M+FU group, indicating that the combination of DP and FU could enhance anti-tumor immune responses in the tumor microenvironment, promote the activation of dendritic cells and macrophages, inhibit tumor growth, improve the tumor microenvironment, and increase survival rates in mice [74]. A study has proved that IFN-γ could inhibit the growth of subcutaneous tumors in mice by directly inhibiting tumor metabolism and inducing tumor cell apoptosis [75]. By activating immune cells (such as CD8^+^ T cells and NK cells), it directly inhibited the proliferation of tumor cells. In vitro experiments showed that IFN-γ stimulation significantly inhibited the proliferation of CT26 colon cancer cells, while in vivo experiments demonstrated that IFN-γ stimulation significantly suppressed tumor growth in immunodeficient mice [76]. Our experiment results showed that, compared with the CON group, serum IFN-γ levels were increased in all treatment groups of mice with colorectal tumors. Among them, there was a significant difference between the FU group (1009.74 ± 241.38 pg/mL) and the CON group (700.23 ± 134.52 pg/mL).

IL-6 is a multifunctional cytokine with a wide range of biological effects, which may serve as a useful marker for predicting poor prognosis in patients with colorectal cancer and may represent a potential therapeutic target for colorectal cancer [77]. High IL-6 levels were typically associated with a higher tumor burden, poorer immune status and faster disease progression [78]. In this experiment, the IL-6 levels in the four groups of tumor-bearing mice were 364.61 ± 35.89, 347.16 ± 40.34, 355.84 ± 31.07, and 345.64 ± 28.23 pg/mL, respectively. Among these, the Pre.DP-M+FU group had the lowest levels, suggesting that the combined DP and FU intervention may inhibit tumor progression by reducing serum IL-6 levels. In the tumor microenvironment of colorectal cancer, the increase in TNF-α level has been considered to play a role in tumor progression and metastasis [79]. In the subcutaneous tumor model of CT26 colon cancer cells, TNF-α secreted by tumor cells can induce the infiltration of myeloid-derived suppressor cells (MDSCs), thus promoting tumor metastasis [80]. This study found that, compared with the CON group, TNF-α levels in the treatment groups decreased by 3.66%, 5.85%, and 6.76%, respectively, indicating that the combined DP and FU intervention could inhibit tumor metastasis.

In summary, DP exerted anti-colorectal tumor effects by modulating cytokines, balancing the immune response, inhibiting tumor cell proliferation and differentiation, and improving the tumor microenvironment, thereby reducing tumor resistance to FU.

## 4. Conclusions

In this study, a CT26 colorectal tumor xenograft mouse model was established to evaluate the effects of different DP doses, intervention modes, and their combined use with FU in tumor-bearing mice. The results showed that low and medium doses of DP increased the spleen index and partly alleviated FU-induced immune organ atrophy, renal and cardiac toxicity, intestinal injury, and muscle wasting. DP also attenuated FU-induced myelosuppression, as evidenced by increased lymphocyte and monocyte levels, reduced granulocyte levels, inhibition of tumor metastasis, and promotion of platelet production, thereby improving tumor-induced hematopoietic suppression. It is worth mentioning that the pre-intervention strategy produced better effects. In terms of immunoregulation, DP promoted the proliferation of CD4^+^ and CD8^+^ T cells, induced the differentiation of naïve T cells into Tem cells, enhanced the generation of Tcm cells, and counteracted chemotherapy-induced immune exhaustion. In addition, DP reduced neutrophil levels, regulated B cell function, and re-established the anti-tumor immune balance. Early intervention also showed a potential advantage in maintaining NK cells. DP further increased the levels of IL-2, IL-10, GM-CSF, and IFN-γ, while decreasing the levels of IL-6 and TNF-α. Overall, this study provided a scientific basis for the application of DP in adjuvant therapy for colorectal cancer. It also laid a theoretical foundation for optimizing nutritional treatment strategies for colorectal cancer and for developing dual-protein foods for special medical purposes.

However, it should be noted that the pre-intervention groups received DP for a total of 38 days (from day 7 to day 45), whereas the direct intervention groups received DP for only 17 days (from day 28 to day 45). Therefore, the observed differences between the two intervention schedules may reflect not only the timing of initiation but also the cumulative DP exposure. Future studies with equal total exposure durations are needed to isolate the true effect of pre-intervention per se. Additionally, cage-to-cage variation was not statistically controlled because the cage was not used as an experimental unit. Moreover, the main limitation of this study was that the subcutaneous tumor model could not fully simulate the mucosal immune characteristics of primary colorectal cancer and the interaction of intestinal microflora, so it is necessary to be cautious when extrapolating the research results to clinical patients. Future research should be conducted in models closer to clinicopathological features, such as an orthotopic transplanted tumor model, APC ^Min/+^ transgenic mouse model or patient-derived xenotransplantation model.

## Figures and Tables

**Table 1:** Dosage and experimental grouping (*n* = 8).

Group	Dosage	DP Pre-Intervention	Abbreviation
Control group	Sterile water	×	CON
Dual-protein groups	0.33 g/kg	√	Pre.DP-L
		×	DP-L
	0.67 g/kg	√	Pre.DP-M
		×	DP-M
	1.33 g/kg	√	Pre.DP-H
		×	DP-H
5-Fluorouracil group	20 mg/kg	×	FU
Composite treatment group	0.67 g/kg DP + 20 mg/kg FU	√	Pre.DP-M+FU
		×	DP-M+FU

**Table 2:** Estimated daily intake of selected nutrients from maintenance feed only and from additional 0.67 g/kg DP gavage, with total intake and percentage change relative to feed only.

Parameter	Feed Only	Additional by Gavage	Total Intake	Change Rate (Relative to Feed Only)
Protein	0.9165 g	+0.0070 g	0.9235 g	0.77%
Fat	0.25 g	+0.0009 g	0.2509 g	0.36%
Carbohydrate	2.5 g	+0.0029 g	~2.5029 g	0.12%
Dietary fiber	0.155 g	+0.0013 g	0.1563 g	0.84%
Calcium	54.5 mg	+0.0536 mg	54.5536 mg	0.10%
Sodium	5 mg	+0.1072 mg	5.1072 mg	2.14%
Vitamin D	0.1675 μg (6.7 IU)	+0.0002 μg	0.1677 μg	0.12%
Vitamin B6	0.03 mg	+0.0003 mg	0.0303 mg	1.00%

**Table 3:** Weight changes of tumor-bearing mice in each group (*n* = 8). Data are expressed as mean ± SD.

Body Weight (g)	CON	Pre. DP-L	DP-L	Pre. DP-M	DP-M	Pre. DP-H	DP-H	FU	Pre.DP-M+FU	DP-M+FU
d 7	19.5 ± 0.6 ^a^	19.6 ± 0.4 ^a^	19.3 ± 0.4 ^a^	18.9 ± 0.5 ^a^	19.0 ± 0.4 ^a^	19.5 ± 0.4 ^a^	19.4 ± 0.5 ^a^	19.4 ± 0.2 ^a^	18.9 ± 0.1 ^a^	19.5 ± 0.6 ^a^
d 10	20.4 ± 0.4 ^a^	20.2 ± 0.5 ^a^	19.7 ± 0.6 ^a^	19.8 ± 0.9 ^a^	19.6 ± 0.5 ^a^	20.1 ± 0.5 ^a^	19.9 ± 0.7 ^a^	19.8 ± 0.2 ^a^	19.1 ± 0.4 ^a^	19.8 ± 0.5 ^a^
d 13	20.6 ± 0.4 ^a^	20.6 ± 0.4 ^ab^	20.0 ± 0.3 ^ab^	20.0 ± 0.7 ^ab^	19.9 ± 0.6 ^ab^	20.4 ± 0.4 ^ab^	20.4 ± 0.5 ^ab^	19.9 ± 0.1 ^b^	19.3 ± 0.3 ^ab^	20.0 ± 0.4 ^ab^
d 16	21.0 ± 0.5 ^a^	20.9 ± 0.3 ^a^	20.4 ± 0.3 ^abc^	20.5 ± 0.7 ^abc^	20.2 ± 0.4 ^abc^	20.7 ± 0.3 ^ab^	20.6 ± 0.5 ^abc^	20.0 ± 0.2 ^bc^	19.6 ± 0.2 ^c^	20.3 ± 0.5 ^abc^
d 19	21.2 ± 0.6 ^a^	21.0 ± 0.3 ^a^	20.6 ± 0.4 ^a^	20.8 ± 0.9 ^a^	20.6 ± 0.4 ^a^	20.8 ± 0.1 ^a^	20.6 ± 0.3 ^a^	20.4 ± 0.2 ^a^	20.1 ± 0.4 ^a^	20.7 ± 0.5 ^a^
d 21	21.2 ± 0.8 ^a^	21.1 ± 0.4 ^a^	20.7 ± 0.4 ^a^	20.8 ± 0.6 ^a^	20.7 ± 0.3 ^a^	20.9 ± 0.2 ^a^	20.9 ± 0.4 ^a^	20.8 ± 0.2 ^a^	20.4 ± 0.4 ^a^	21.2 ± 0.6 ^a^
d 24	21.1 ± 0.8 ^a^	21.2 ± 0.5 ^a^	20.8 ± 0.4 ^a^	21.2 ± 0.4 ^a^	20.9 ± 0.3 ^a^	21 ± 0.2 ^a^	21 ± 0.3 ^a^	20.9 ± 0.1 ^a^	20.7 ± 0.1 ^a^	21.4 ± 0.3 ^a^
d 27	21.4 ± 0.7 ^a^	21.3 ± 0.5 ^a^	20.9 ± 0.4 ^a^	21.5 ± 0.3 ^a^	21.1 ± 0.3 ^a^	21.1 ± 0.1 ^a^	21.2 ± 0.3 ^a^	21.2 ± 0.5 ^a^	20.9 ± 0.2 ^a^	21.4 ± 0.3 ^a^
d 30	23.6 ± 0.4 ^a^	22.8 ± 1.1 ^ab^	23.1 ± 0.4 ^ab^	22.8 ± 0.3 ^ab^	23 ± 0.2 ^ab^	23.0 ± 0.4 ^ab^	23.0 ± 0.1 ^ab^	22.8 ± 1.8 ^ab^	22.7 ± 0.1 ^b^	23.0 ± 0.2 ^ab^
d 33	24.8 ± 0.7 ^a^	23.6 ± 1.9 ^a^	24.1 ± 0.9 ^a^	24.5 ± 0.7 ^a^	24.7 ± 0.7 ^a^	24.6 ± 0.3 ^a^	24.4 ± 1.1 ^a^	24.0 ± 2.0 ^a^	24.4 ± 1.4 ^a^	24.4 ± 0.6 ^a^
d 38	27.2 ± 1.2 ^a^	25.8 ± 2.3 ^a^	26.0 ± 1.7 ^a^	26.8 ± 1.5 ^a^	27.9 ± 1.7 ^a^	25.9 ± 2.3 ^a^	26.9 ± 1.8 ^a^	25.0 ± 1.4 ^a^	24.4 ± 3 ^a^	25.1 ± 3.3 ^a^
d 40	26.9 ± 1.6 ^a^	26.0 ± 2.3 ^a^	26.6 ± 1.8 ^a^	27.1 ± 2 ^a^	28.3 ± 1.3 ^a^	25.7 ± 2.8 ^a^	27.3 ± 2.2 ^a^	25.2 ± 1.5 ^a^	24.6 ± 2.8 ^a^	25.2 ± 3.3 ^a^
d 42	27.2 ± 1.3 ^a^	26.8 ± 2.7 ^ab^	27 ± 2.1 ^ab^	27.3 ± 1.6 ^ab^	29.4 ± 2.1 ^a^	25.8 ± 3 ^ab^	28.3 ± 2.3 ^a^	23.0 ± 0.9 ^b^	25.6 ± 2.8 ^ab^	26.8 ± 2.6 ^ab^
d 45	27.7 ± 1.6 ^a^	27.6 ± 2.7 ^a^	27.2 ± 2.3 ^a^	27.8 ± 2.6 ^a^	30.0 ± 2.7 ^a^	25.3 ± 2.4 ^a^	29.0 ± 2.5 ^a^	22.7 ± 2.4 ^a^	27.0 ± 1.9 ^a^	27.8 ± 2.3 ^a^

Note: Different letters in each row in the table indicate significant differences (*p* < 0.05).

**Table 4:** Changes of tumor volume of mice in each group (*n* = 8). Data are expressed as mean ± SD.

Tumor Volume	d28 (mm^3^)	d30 (mm^3^)	d33 (mm^3^)	d35 (mm^3^)	d38 (cm^3^)	d40 (cm^3^)	d42 (cm^3^)	d45 (cm^3^)
CON	32.8 ± 3.7 ^a^	47± 5 ^a^	67 ± 12 ^a^	211 ± 49 ^a^	0.59± 0.11 ^a^	0.76 ± 0.21 ^a^	0.95 ± 0.23 ^a^	1.59 ± 0.24 ^a^
Pre.DP-L	30.2 ± 3.8 ^a^	48± 4 ^a^	63± 2 ^a^	177 ± 15 ^a^	0.57± 0.17 ^a^	0.78 ± 0.27 ^a^	0.91 ± 0.29 ^a^	1.38 ± 0.59 ^a^
DP-L	29.3 ± 2.8 ^a^	47 ± 10 ^a^	76 ± 18 ^a^	206 ± 82 ^a^	0.54 ± 0.26 ^a^	0.76 ± 0.38 ^a^	0.89 ± 0.49 ^a^	1.47 ± 0.85 ^a^
Pre.DP-M	28.3 ± 4.8 ^a^	50 ± 10 ^a^	88 ± 20 ^a^	211 ± 113 ^a^	0.57 ± 0.36 ^a^	0.82 ± 0.52 ^a^	0.93 ± 0.53 ^a^	1.32 ± 0.81 ^a^
DP-M	30.5 ± 6.0 ^a^	48 ± 13 ^a^	78 ± 20 ^a^	191 ± 86 ^a^	0.48 ± 0.20 ^a^	0.67 ± 0.28 ^a^	0.80 ± 0.31 ^a^	1.38 ± 0.56 ^a^
Pre.DP-H	32.4 ± 1.5 ^a^	56 ± 7 ^a^	87 ± 32 ^a^	210 ± 131 ^a^	0.51 ± 0.28 ^a^	0.64 ± 0.34 ^a^	0.90 ± 0.31 ^a^	1.23 ± 0.21 ^a^
DP-H	32.7 ± 1.5 ^a^	60± 14 ^a^	97.5 ± 26.6 ^a^	255 ± 107 ^a^	0.65 ± 0.31 ^a^	0.80 ± 0.38 ^a^	0.96 ± 0.42 ^a^	1.41 ± 0.65 ^a^
FU	28.4 ± 4.6 ^a^	60± 27 ^a^	117.5 ± 46.5 ^a^	314 ± 105 ^a^	0.64 ± 0.24 ^a^	0.65 ± 0.23 ^a^	0.75 ± 0.38 ^a^	1.03 ± 0.80 ^a^
Pre.DP-M+FU	28.9 ± 4.5 ^a^	45± 14 ^a^	84.9 ± 30.5 ^a^	191 ± 90 ^a^	0.59 ± 0.26 ^a^	0.66 ± 0.35 ^a^	0.85 ± 0.42 ^a^	1.18 ± 0.39 ^a^
DP-M+FU	28.7 ± 3.4 ^a^	49± 18 ^a^	124.6 ± 51.6 ^a^	271 ± 129 ^a^	0.57 ± 0.29 ^a^	0.65 ± 0.26 ^a^	0.87 ± 0.48 ^a^	1.37 ± 0.91 ^a^

Note: Different letters in each column in the table indicate significant differences (*p* < 0.05).

**Table 5:** Tumor weight and tumor inhibition rate in each group of mice (*n* = 8). Data are expressed as mean ± SD.

Group	Tumor Weight (g)	Tumor Inhibition Rate (%)
CON	1.5999 ± 0.6275	-
Pre.DP-L	1.4811 ± 0.6911	7.43%
DP-L	1.5032 ± 0.8891	6.05%
Pre.DP-M	1.3534 ± 0.9539	15.41%
DP-M	1.4914 ± 0.1664	6.78%
Pre.DP-H	1.3712 ± 0.9134	14.3%
DP-H	1.5418 ± 0.8756	3.63%
FU	0.9716 ± 0.6990	39.27%
Pre.DP-M+FU	1.1282 ± 0.4476	29.49%
DP-M+FU	1.1409 ± 0.5213	28.69%

## Data Availability

The original contributions presented in this study are included in the article/Supplementary Material. Further inquiries can be directed to the corresponding author or first author.

## Supplementary Materials

The following supporting information can be downloaded at: https://www.mdpi.com/article/10.3390/nu18111663/s1, Table S1: Table of nutrient list of dual-protein complex nutrition powder; Table S2: Amino acid composition in dual-protein complex nutrition powder; Table S3: Test results of Rat and Mouse maintenance feed; Table S4: Nutritional composition of the standard rodent diet for inbred strains; Figure S1: Representative images of tumors from each group.

## Abbreviations

The following abbreviations are used in this manuscript:

ALB	Albumin
CK	Creatine kinase
ALP	Alkaline phosphatase
ALT	Alanine aminotransferase
AST	Aspartate aminotransferase
CRC	Colorectal cancer
DP	Dual- protein
FU	5-Fluorouracil
GM-CSF	Granulocyte–macrophage colony-stimulating factor
HDL-C	High-density lipoprotein cholesterol
HGB	Hemoglobin
IFN-γ	Interferon-gamma
IL-10	Interleukin-10
IL-2	Interleukin-2
IL-4	Interleukin-4
IL-6	Interleukin-6
LDH	Lactate dehydrogenase
LDL-C	Low-density lipoprotein cholesterol
LYM	Lymphocyte
MON	Monocytes
NK	Natural killer
PLT	Platelets
RBC	Red blood cell
GRA	Granulocytes
TC	Total cholesterol
TG	Triglycerides
TNF-α	Tumor necrosis factor-alpha
TP	Total protein
WBC	White blood cell
