# Supplementary material for: Dual-Protein Intervention in CT26 Tumor-Bearing Mice: A Preliminary Evaluation of Its Effects on Anti-Tumor Efficacy of 5-Fluorouracil and Immune Responses
Source: Nutrients. 2026 May 22;18(11):1663. doi: 10.3390/nu18111663 (PMC13258669; doi:10.3390/nu18111663)
Supplement: Supplementary file 1 [file nutrients-18-01663-s001.zip › nutrients-4262234-supplementary.pdf]

Table S1. Table of nutrient list of dual-protein complex nutrition powder

Nutrients	Per 100g	NRV%
Energy	1579 KJ	19%
Protein	52.5 g	88%
Fat	6.5 g	11%
Carbohydrates	21.5 g	7%
Dietary fiber	10.0 g	40%
Sodium	800 mg	40%
Vitamin D	1.5 µg	30%
Vitamin B6	2.00 mg	143%
Calcium	400 mg	50%

Note: Nutrient Reference Values (NRV).

Table S2. Amino acid composition in dual-protein complex nutrition powder

Composition	Results (g/100g)	Composition	Results (g/100g)
Aspartic acid	4.92	Leucine*	4.17
Threonine*	2.58	Isoleucine*	2.21
Serine	2.35	Methionine*	0.87
Glutamic acid	9.20	Phenylalanine*	2.63
Proline	3.42	Lysine*	1.47
Glycine	1.58	Histidine	1.18
Alanine	2.69	Tryptophan*	0.55
Cysteine	0.45	Arginine	3.02
Valine*	2.57	Tyrosine	1.47
EAA content	19.15	BCAA	8.95
NEAA content	30.25	Total 18 Amino Acids	49.4

Note: * indicates essential amino acids.

Table S3. Test results of Rat and Mouse maintenance feed

Test Item	Result	Test Item	Result	Test Item	Result
Total Arsenic (calculated on 88% dry matter basis)	0.32 mg/kg	Arginine (calculated on 90% dry matter basis)	11.1 g/kg	Choline (calculated on 90% dry matter basis)	1335 mg/kg
Lead (calculated on 88% dry matter basis)	0.096 mg/kg	Histidine (calculated on 90% dry matter basis)	4.2 g/kg	Magnesium (calculated on 90% dry matter basis)	2.2 g/kg

Cadmium (calculated on 88% dry matter basis)	0.052 mg/kg	Tryptophan (calculated on 90% dry matter basis)	2.2 g/kg	Potassium (calculated on 90% dry matter basis)	8.94 g/kg
Mercury (calculated on 88% dry matter basis)	Not detected (quantitation limit: 0.01 mg/kg)	Phenylalanine + Tyrosine (calculated on 90% dry matter basis)	12.1 g/kg	Sodium (calculated on 90% dry matter basis)	2.11 g/kg
BHC (calculated on 88% dry matter basis)	Not detected (detection limit: 0.001 mg/kg)	Threonine (calculated on 90% dry matter basis)	6.5 g/kg	Iron (calculated on 90% dry matter basis)	1.2×10^2 mg/kg
DDT (calculated on 88% dry matter basis)	Not detected (detection limit: 0.001 mg/kg)	Leucine (calculated on 90% dry matter basis)	15.0 g/kg	Manganese (calculated on 90% dry matter basis)	1.1×10^2 mg/kg
Aflatoxin B1 (calculated on 88% dry matter basis)	0.170 μg/kg	Isoleucine (calculated on 90% dry matter basis)	7.6 g/kg	Copper (calculated on 90% dry matter basis)	20 mg/kg
Aerobic Plate Count	<10 cfu/g	Valine (calculated on 90% dry matter basis)	8.4 g/kg	Zinc (calculated on 90% dry matter basis)	58 mg/kg
Coliforms	<30 MPN/100g	Vitamin A (calculated on 90% dry matter basis)	8.17×10^3 IU/kg	Selenium (calculated on 90% dry matter basis)	0.13 mg/kg
Molds and Yeasts	<10 cfu/g	Vitamin D3 (calculated on 90% dry matter basis)	1.34×10^3 IU/kg	Iodine	0.534 mg/kg
Salmonella	Not detected	Vitamin E (calculated on 90% dry matter basis)	119 IU/kg	Aspartic Acid	1.48%
Moisture and Other Volatile Matter	84 g/kg	Vitamin K3 (calculated on 90% dry matter basis)	6.4 mg/kg	Serine	0.82%
Crude Protein (calculated on 90% dry matter basis)	183.3 g/kg	Vitamin B1 (calculated on 90% dry matter basis)	13.4 mg/kg	Glutamic Acid	3.19%
Crude Fat (calculated on 90%	50 g/kg	Vitamin B2 (calculated on 90%	10.3 mg/kg	Proline	0.92%

dry matter basis)		dry matter basis)			
Crude Fiber (calculated on 90% dry matter basis)	31 g/kg	Vitamin B6 (calculated on 90% dry matter basis)	9.37 mg/kg	Glycine	0.75%
Crude Ash (calculated on 90% dry matter basis)	64 g/kg	Niacin (calculated on 90% dry matter basis)	65.0 mg/kg	Alanine	0.89%
Calcium (calculated on 90% dry matter basis)	10.9 g/kg	Pantothenic Acid (calculated on 90% dry matter basis)	30.2 mg/kg	Methionine (calculated on 90% dry matter basis)	0.37%
Total Phosphorus (calculated on 90% dry matter basis)	8.2 g/kg	Folic Acid (calculated on 90% dry matter basis)	6.52 mg/kg	Phenylalanine (calculated on 90% dry matter basis)	0.75%
Lysine (calculated on 90% dry matter basis)	8.8 g/kg	Biotin (calculated on 90% dry matter basis)	0.176 mg/kg	Cystine (calculated on 90% dry matter basis)	0.30%
Methionine + Cystine (calculated on 90% dry matter basis)	6.7 g/kg	Vitamin B12 (calculated on 90% dry matter basis)	0.033 mg/kg	Tyrosine (calculated on 90% dry matter basis)	0.46%

Table S4. Nutritional composition of the standard rodent diet for inbred strains

Parameter	Results	Parameter	Results
Moisture	98 g/kg	Calcium	11 g/kg
Crude protein	234 g/kg	Total phosphorus	7.2 g/kg
Crude fat	51 g/kg	Aflatoxin B1	Not detected
Crude fiber	39 g/kg	Total bacterial count	＜10 CFU/g
Crude ash	63 g/kg		

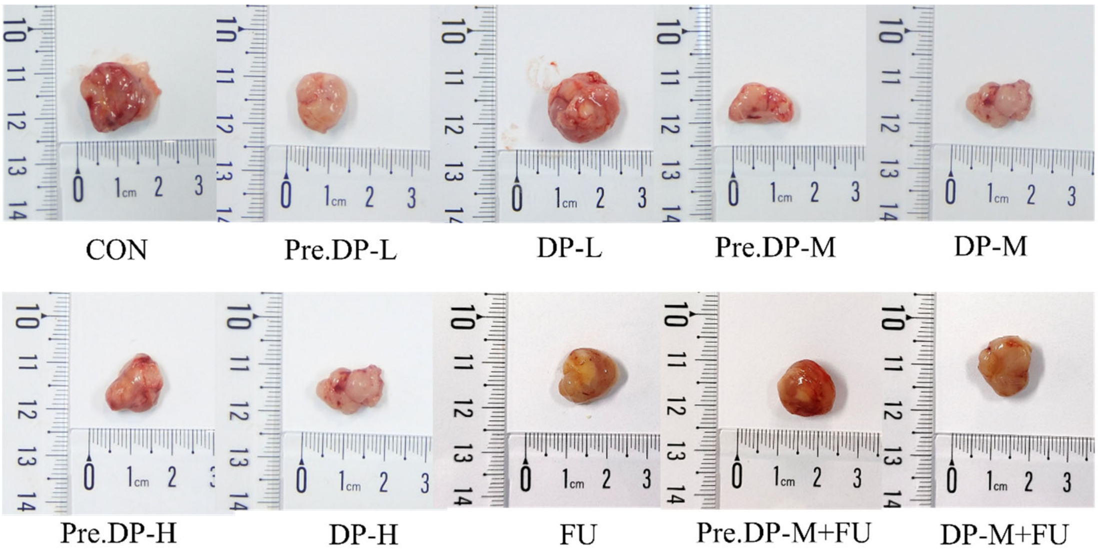

Figure S1. Representative images of tumors from each group
